# Radiosynthesis and Preclinical Evaluation of an ^18^F-Labeled Triazolopyridopyrazine-Based Inhibitor for Neuroimaging of the Phosphodiesterase 2A (PDE2A)

**DOI:** 10.3390/ph15101272

**Published:** 2022-10-15

**Authors:** Barbara Wenzel, Stefan R. Fritzsche, Magali Toussaint, Detlef Briel, Klaus Kopka, Peter Brust, Matthias Scheunemann, Winnie Deuther-Conrad

**Affiliations:** 1 Department of Neuroradiopharmaceuticals, Institute of Radiopharmaceutical Cancer Research, Helmholtz-Zentrum Dresden-Rossendorf, 04318 Leipzig, Germany; 2 Institute for Drug Discovery, Faculty of Medicine, Leipzig University, 04103 Leipzig, Germany; 3 Faculty of Chemistry and Food Chemistry, School of Science, Technical University Dresden, 01069 Dresden, Germany

**Keywords:** PDE2A inhibitor, triazolopyridopyrazine, fluorine-18, small animal PET-MR, autoradiography, in vivo metabolism

## Abstract

The cyclic nucleotide phosphodiesterase 2A is an intracellular enzyme which hydrolyzes the secondary messengers cAMP and cGMP and therefore plays an important role in signaling cascades. A high expression in distinct brain areas as well as in cancer cells makes PDE2A an interesting therapeutic and diagnostic target for neurodegenerative and neuropsychiatric diseases as well as for cancer. Aiming at specific imaging of this enzyme in the brain with positron emission tomography (PET), a new triazolopyridopyrazine-based derivative (**11**) was identified as a potent PDE2A inhibitor (IC_50,_ _PDE2A_ = 1.99 nM; IC_50, PDE10A_ ~2000 nM) and has been radiofluorinated for biological evaluation. In vitro autoradiographic studies revealed that **[^18^F]11** binds with high affinity and excellent specificity towards PDE2A in the rat brain. For the PDE2A-rich region nucleus caudate and putamen an apparent *K*_D_ value of 0.24 nM and an apparent *B*_max_ value of 16 pmol/mg protein were estimated. In vivo PET-MR studies in rats showed a moderate brain uptake of **[^18^F]11** with a highest standardized uptake value (SUV) of 0.97. However, no considerable enrichment in PDE2A-specific regions in comparison to a reference region was detectable (SUV_caudate putamen_ = 0.51 vs. SUV_cerebellum_ = 0.40 at 15 min p.i.). Furthermore, metabolism studies revealed a considerable uptake of radiometabolites of **[^18^F]11** in the brain (66% parent fraction at 30 min p.i.). Altogether, despite the low specificity and the blood–brain barrier crossing of radiometabolites observed in vivo, **[^18^F]11** is a valuable imaging probe for the in vitro investigation of PDE2A in the brain and has potential as a lead compound for further development of a PDE2A-specific PET ligand for neuroimaging.

## 1. Introduction

The 3′,5′-cyclic nucleotide phosphodiesterase 2A (PDE2A) belongs to a superfamily of highly conserved enzymes (PDE1-11). These omnipresent PDEs are the only enzymes degrading the secondary messengers cAMP and cGMP, thereby terminating the intracellular signalling pathways addressed by G-protein coupled receptors (GPCRs) as well as by natriuretic peptides and nitric oxide, respectively. The high relevance of cyclic nucleotide signalling for physiological processes makes PDEs in general a choice for exploration as therapeutic targets for the treatment of numerous pathophysiological conditions. Furthermore, because of isoform specific expression profiles, PDEs are also considered biomarkers for certain diseases of, e.g., the nervous, cardiovascular, and immune systems as well as of cancer [1].

The specific configuration of the substrate-binding pocket determines the dual substrate specificity of PDE2A [2], which is mainly expressed in the brain, in particular in the dorsal striatum, frontal cortex, and hippocampus, as well as in the heart, liver, lung, adrenal gland, and bladder [1,3,4]. Given the involvement of striatal cyclic nucleotides in the GPCR-mediated dopamine signalling, selective PDE2A inhibitors may have the potential to alleviate impairments associated with schizophrenia [5,6]. However, despite promising preclinical results obtained with drug candidates such as PF-05180999 [6] or TAK-915 [7], no clinical efficacy trials have been reported so far. Importantly, a high expression of PDE2A has been detected in cancer cells, e.g., in squamous carcinoma HeLa cells [8] and oral osteosarcoma HOSM-1 cells [9] along with cytotoxic and pro-apoptotic effects of pharmacological inhibition of PDEs [8,9]. These findings together with the endothelial expression of PDE2A in combination with the relevance of cAMP levels for angiogenesis [3,10] make PDE2A a potential therapeutic and diagnostic target also in the context of cancer. On the other hand, there is evidence for a lower expression in liver carcinoma compared with healthy tissue [11] and for an inhibitory role of PD2A in gliomagenesis [12].

Non-invasive imaging of potential drug targets and biomarkers such as PDE2A by means of PET may help in analysing the relationship between PDE2A protein level and tumor biology and is suggested to increase the success rate in PDE2A targeted drug discovery. Having available a brain penetrant and PDE2A specific ligand labelled with a positron emitting radionuclide such as fluorine-18, patients eligible for brain PDE2A-targeted therapeutic trials could be identified as well as the target occupancy of potential therapeutics could be investigated. Furthermore, in preclinical research longitudinal and dynamic studies in appropriate animal models on the role of the cGMP/cAMP-PDE2A pathways in various diseases would be enabled.

So far, all intentions to develop PDE2A specific radiopharmaceuticals are on the basis of PDE-targeted therapeutics with low-molecular weight mainly acting as inhibitors via binding to the substrate binding pocket at the catalytic site of the enzymes [1,13,14]. In brief, despite numerous efforts during the last years, the imidazolotriazine derivative [^18^F]PF-05270430 remains so far the most advanced tracer with preclinical and first in human studies reported already in 2016 [15,16]. However, a low binding potential along with the presence of radiometabolites in the brain significantly limits the applicability of this radioligand [16,17] so that currently, no clinically validated PET tracer exists for the quantitative imaging of PDE2A.

**Scheme 1:**
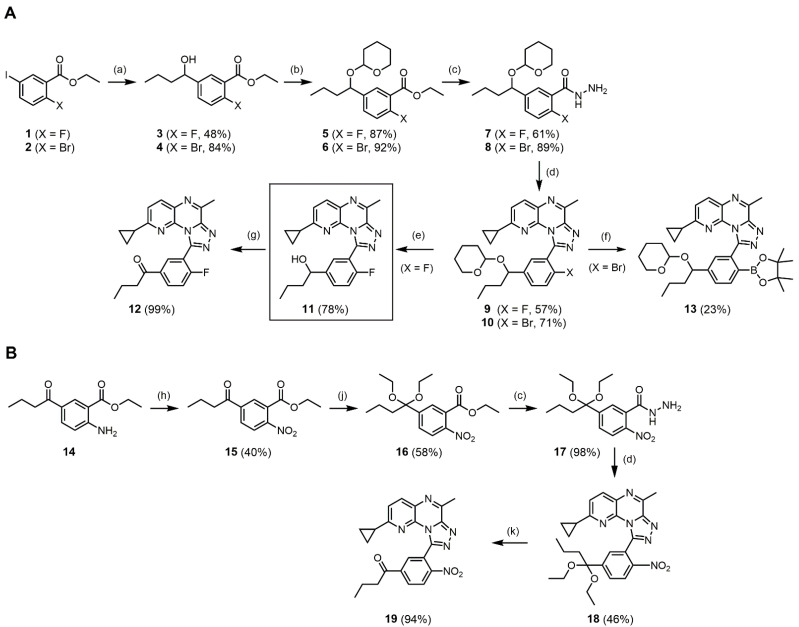
Synthesis of the PDE2A inhibitor **11**, the boronic acid pinacol ester precursor **13** (**A**) and the nitro precursor **19** (**B**). Reaction conditions: (**a**) (i) 1.1 eq *i*PrMgCl · LiCl, (ii) 1.2 eq butanal, tetrahydrofuran (THF), −40 to −50 °C, (**b**) 1.2 eq 3,4-dihydropyran, 1 mol% TsOH · H_2_O, dichloromethane (DCM), rt, (**c**) 10.0 eq N_2_H_4_ · H_2_O, MeOH, (**d**) (i) 3-chloro-6-cyclopropyl-2-methylpyrido[2,3-*b*]pyrazine [20], 6.0 eq DBU, acetonitrile (ACN), rt, 5 h, (ii) 2.0 eq PPh_3_, 2.0 eq C_2_Cl_6_, rt, (**e**) 10 mol% PPTS, MeOH, rt, (**f**) 2 × 1.1 eq (bispinacolato)diborane (B_2_pin_2_), 2 × 5 mol% Pd(dppf)Cl_2_, 3.0 eq KOAc, dioxane, 80 °C, (**g**) 1.2 eq DMP, DCM, (**h**) 6.0 eq H_2_O_2_, 7.0 eq trifluoroacetic anhydride (TFAA), DCM, rt, (**j**) 1.0 eq HCl, 20.0 eq HC(OEt)_3_, EtOH, reflux, (**k**) 18 mol% TsOH · H_2_O, acetone, rt.

**Scheme 2:**
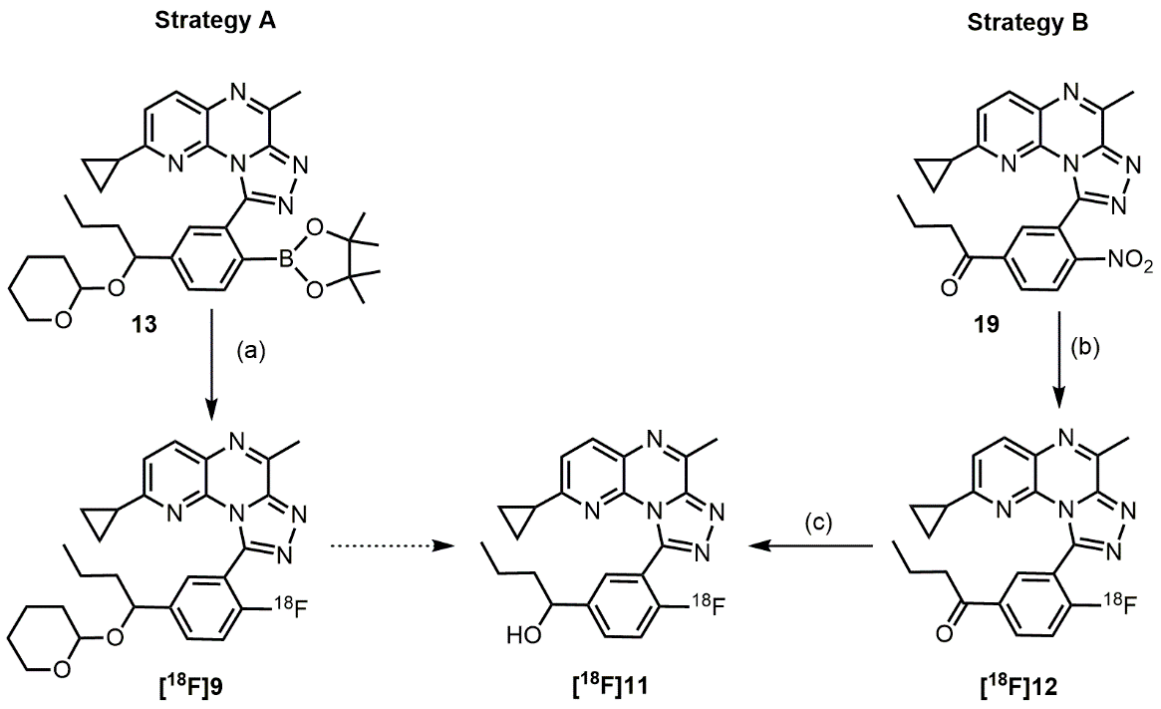
Radiofluorination strategies for the generation of **[^18^F]11**. Reaction conditions: (**a**) shown in Table 1, (**b**) [^18^F]TBAF in ACN, 110 °C, 15 min, (**c**) NaBH_4_/*n*-butanol, 35 °C, 5 min.

In our continuous efforts to develop an ^18^F-labeled ligand for neuroimaging of PDE2A with PET we used the same approach and selected triazolopyridopyrazines as small-molecule lead compounds with inhibitory potency in the low-nanomolar range and high selectivity towards co-localized PDEs. The principle suitability of such tricyclic core structures as potent inhibitors of PDE2A has been already previously proven by our group [18,19] and others [20]. According to docking studies with similar triazolopyrazine-based derivatives, the tricyclic core fits in the hydrophobic clamp of the active site of the PDE2A structure [21,22]. Thus, a series of 12 new fluoro-containing derivatives was synthesized and their potencies as inhibitors against PDE2A and PDE10A were determined [23]. Compound **11** was one of the best inhibitors (Scheme 1A) with an IC_50_ value of 1.99 nM for PDE2A and a nearly 1000-fold selectivity vs. PDE10A (IC_50, PDE10A1_ = 1910 nM). A high selectivity against PDE10A is essential for specific imaging, as both enzymes are partially localized in the same brain regions [24]. As the radiofluorination on the respective non-activated position of the phenyl ring of **11** is not straightforward, two strategies for radio-labeling based on two different precursor compounds were designed (Scheme 2). Finally, we investigated the successfully synthesized radioligand **[^18^F]11** in autoradiographic and pharmacokinetic studies with wildtype rodents.

## 2. Results and Discussion

### 2.1. Organic Syntheses of the New Inhibitor ***11*** and the Precursor Compounds ***13*** and ***19***

Starting from the esters **1** and **2**, the hydroxybutyl sidechain was introduced by metalation with isopropylmagnesium chloride lithium chloride complex (*i*PrMgCl · LiCl), followed by a Grignard reaction with butanal using a modified procedure from Giovannini et al. [20] (Scheme 1A). Subsequently, the hydroxy group was protected as 2-tetrahydropyranyl ether (2-THP) and the resulting esters **5** and **6** were converted into the benzohydrazides **7** and **8**. Cyclization with 3-chloro-6-cyclopropyl-2-methylpyrido[2,3-*b*]pyrazine [20] was accomplished by the use of 1,8-diazabicyclo[5.4.0]undec-7-en (DBU) as a base for hydrazide activation [25] followed by the addition of PPh_3_/C_2_Cl_6_ as a reagent for dehydration and ring closure to the triazole [26] in a one-pot reaction at room temperature. Deprotection of the resulting fluoro-derivative **9** with pyridinium *p*-toluenesulfonate (PPTS) yielded the new inhibitor **11**. Oxidation of the secondary alcohol with Dess–Martin periodinane (DMP) produced compound **12** which was needed as an intermediate reference for the radiosynthesis. Miyaura borylation [27] of the bromo derivative **10** yielded the boronic acid pinacol ester **13**, one of the two different radiosynthesis precursor compounds. The synthesis of the other precursor started with the oxidation of **14** [28] to the corresponding nitrosubstituted compound **15** using a modified procedure from Hahm et al. [29] (Scheme 1B). Cyclization to the triazolo compound **18** was accomplished after the protection of the ketone as the ketal **16** and conversion of the ester to the hydrazide **17**. The final deprotection with *p*-toluenesulfonic acid (TsOH) yielded the nitro precursor **19**.

### 2.2. Radiosyntheses

As depicted in Scheme 2, two strategies have been drafted for the generation of **[^18^F]11**. While with strategy A a copper-mediated radiofluorination (CMRF) was envisaged, strategy B is based on a common nucleophilic aromatic substitution reaction using a carbonyl function in *para*-position for activation of the labeling position. With the deprotection (strategy A) and reduction (strategy B), respectively, a second reaction step was necessary for both procedures.

#### 2.2.1. Copper-Mediated Radiofluorination Experiments-Strategy A

The attempts to synthesize the intermediate **[^18^F]9** by CMRF have been performed with a boronic acid pinacol ester (**13**) as a precursor (Scheme 2) under different reaction conditions adopted from the literature [30,31,32,33,34,35] (see Table 1). In most of the reported procedures rather high amounts of precursor (30–60 µmol) are used which is however sometimes not practicable as for example in our study due to an elaborated organic precursor synthesis. Generally, for the here described experiments only 2 mg (3.4 µmol) of the protected precursor **13** were used and the labeling reactions were conducted at temperatures ranging from 110–140 °C with [Cu(OTf)_2_(py)_4_] as copper catalyst. For monitoring of the labeling progress, aliquots were taken every 5 min (total reaction time 20 min) and analysed by radio-TLC. In the first labeling experiments, [^18^F]tetra-*n*-butylammonium fluoride ([^18^F]TBAF, generated from [^18^F]fluoride and (*n*-Bu)_4_NHCO_3_ under azeotropic drying) in *N,N*-dimethylacetamide (DMA)/*tert*-butanol (*tert*-BuOH) with a precursor-to-copper catalyst ratio of 1:3.5 (entry 1 in Table 1) was chosen as these conditions have been shown to be efficient for the labeling of several radiotracers in our lab [36,37]. However, an RCY of only 0.6% could be achieved which prompted us to test *n*-butanol as probably a more efficient alcohol additive [32] without achieving an improvement. Furthermore, the reduction of the content of [Cu(OTf)_2_(py)_4_] to a ratio of precursor-to-copper catalyst of 1:1 did not increase the fraction of labeling product (entry 2 in Table 1). Moreover, the use of a higher amount of precursor (8.5 µmol, entry 4) did not give higher labeling yields. In order to exclude a methodical failure of the labeling procedures, a standard labeling system was established using 4-biphenylboronic acid pinacol ester and [Cu(OTf)_2_(py)_4_]. As shown in the right column of Table 1, the synthesized 4-[^18^F]fluorobiphenyl could be produced in sufficient amounts under the selected reaction conditions.

In the next step, the conventional potassium carbonate-kryptofix-222 system [^18^F]F^−^/K_222_/K_2_CO_3_ (generated by elution of [^18^F]fluoride from an anion-exchange cartridge with K_2_CO_3_, addition of K_222_ and azeotropic drying) was used as fluorination agent with *N,N*-dimethylformamide (DMF) as solvent. However, no product formation could be observed at precursor-to-copper catalyst ratios of 1:1.5 and 5:1 (entries 5 and 6).

To finally investigate a CMRF system with a low base content, the fluorination agent [^18^F]4-(dimethylamino)pyridinium fluoride ([^18^F]DMAPF) was synthesized by elution of ^18^F-fluoride from the anion exchange cartridge with 4-(dimethylamino)pyridinium trifluoromethanesulfonate (DMAPOTf) in methanol and subsequent evaporation of the alcohol [35,38]. While high RCYs could be obtained for the standard labeling reaction of [^18^F]4-fluorobiphenyl in DMA and 1,3-dimethyl-2-imidazolidinone (DMI), less than 1% has been found for **[^18^F]9** (entries 7–9).

As already discussed by the group of Gouverneur, late-stage ^18^F-incorporation by CMRF can bear risks, in particular when the compound to be radiolabeled contains *N*-heterocycles [39,40]. Thus, the ^18^F-fluorination might be inhibited due to a complexation of copper by the lewis base nitrogen atoms generating non-reactive copper species. To investigate the potential influence of the *N*-heterotricyclic triazolopyridopyrazine core on the labeling process, we performed a test reaction with the standard labeling system (4-biphenylboronic acid pinacol ester) under the addition of an equimolar amount of **12**. The obtained RCY of 60% indicates, that the *N*-heterocycles do not prevent the formation of reactive copper-fluorination species. Based on this result, we assume that the unsuccessful radiofluorination of **[^18^F]9** might be caused by a sterical hindrance due to the bulky tricyclic moiety substituted in *ortho*-position to the also space-consuming boronic acid pinacol ester leaving group of the phenyl ring and thus preventing the attack of the Cu(II)-^18^F-complex.

#### 2.2.2. Aromatic Nucleophilic Substitution Reactions-Strategy B

The aromatic nucleophilic substitution reaction of the nitro precursor **19** (Scheme 2) was first investigated with the conventional [^18^F]F^−^/K_222_/K_2_CO_3_ system using different solvents and temperatures (entries 1–3 in Table 2). However, neither the use of acetonitrile (ACN) at 110 °C nor DMF or DMSO at 150 °C resulted in the formation of the intermediate **[^18^F]12**. Inspection of samples of the reaction mixtures with UV-HPLC revealed the entire decomposition of the precursor in all three solvents already after 5 min reaction time. Therefore, we assumed that the precursor might be sensitive to the basic labeling conditions and tested [^18^F]TBAF as a milder nucleophilic ^18^F-fluorination agent. Labeling reactions were performed in ACN, ACN/DMSO, *tert*-butanol and DMF (entries 4–7 in Table 2). While the reaction in *tert*-butanol did not result in the formation of **[^18^F]12**, maximum RCYs of 21% could be achieved with ACN. In contrast to the [^18^F]F^−^/K_222_/K_2_CO_3_ system, intact precursor could be detected in all solvents even after 20 min labeling time. All reactions were performed with small precursor amounts of about 1–2 mg.

To reduce the keton function of **[^18^F]12** in the second reaction step, sodium borhydride (NaBH_4_, 2M solution) with *n*-butanol and lithium aluminum hydride (LiAlH_4_, 1M solution) have been tested as reducing agents by direct addition to the labeling reaction mixture at 35 °C. The reduction was completed already after 5 min reaction time; however, with LiAlH_4_ the formation of a slightly more hydrophilic radioactive byproduct has been observed accounting for about the same amount as the desired product.

Finally, for this two-step one-pot radiolabeling procedure on the basis of the nitro precursor **19**, highest RCYs for the radiofluorination step were achieved with [^18^F]TBAF in ACN at 110 °C after a reaction time of 15 min. For the second step, the reduction system NaBH_4_/*n*-butanol at 35 °C turned out to be most useful for a quantitative conversion of the intermediate **[^18^F]12** to the desired radiotracer **[^18^F]11**.

#### 2.2.3. Automated Radiosynthesis of **[^18^F]11**

The developed reaction conditions were translated to an automated procedure using the TRACERlab FX2 N synthesis module (GE Healthcare). The synthesizer setup is described in the experimental part. In brief, after trapping and elution of [^18^F]fluoride by a solution of *tetra*-n-butylammonium hydrogencarbonate from an anion exchange cartridge, the labeling reaction of the azeotropically dried [^18^F]TBAF with the nitro precursor **19** was performed in ACN at 110 °C for 15 min. For the subsequent reduction step, sodium borhydride in *n*-butanol was added and the reaction proceeded at 35 °C for 5 min. To isolate **[^18^F]11**, the crude reaction mixture was diluted with a solution of aqueous 20 mM ammonium formate, acetonitrile and water and directly applied to a semi-preparative radio-HPLC system. The radiotracer fraction was collected at a retention time of 28–30 min and reformulated by solid phase extraction (SPE) using a C18-cartridge. The long retention time was needed in order to separate the radiotracer from the remaining nitro precursor, whose keto function was also reduced and therefore eluted close in front of the radiotracer (Figure S12 in Supplementary Materials). The obtained radiotracer eluate was transferred out of the hot cell, concentrated and formulated in sterile isotonic saline. Consequently, the formulated product contains 10% of ethanol and had a radiochemical concentration of about 1 MBq/µL. In a total synthesis time of ~80 min, **[^18^F]11** could be produced with a high radiochemical purity of ≥99%, a radiochemical yield of 2.2 ± 0.7% (*n* = 8) and molar activities in the range of 11–20 GBq/µmol (*n* = 4, end of synthesis EOS) with starting activities of 6–8 GBq. The identity of **[^18^F]11** has been confirmed by analytical radio-HPLC of the final product spiked with the reference compound **11** (Figure S13 in Supplementary Materials).

In order to estimate the lipophilicity of **[^18^F]11**, the partition coefficient was determined by the shake flask method using *n*-octanol and phosphate-buffered saline as partition system. With this method a logD value of 1.79 ± 0.66 (*n* = 4) could be determined. The chemical stability of the radiotracer was investigated by incubation in phosphate-buffered saline (PBS) and *n*-octanol at 30 °C. **[^18^F]11** proved to be stable in these media and inspection by radio-HPLC revealed no defluorination or degradation within 60 min of incubation time.

### 2.3. Biological Evaluation

#### 2.3.1. In Vitro Autoradiography with Rat Brain Cryosections

**Figure 1:**
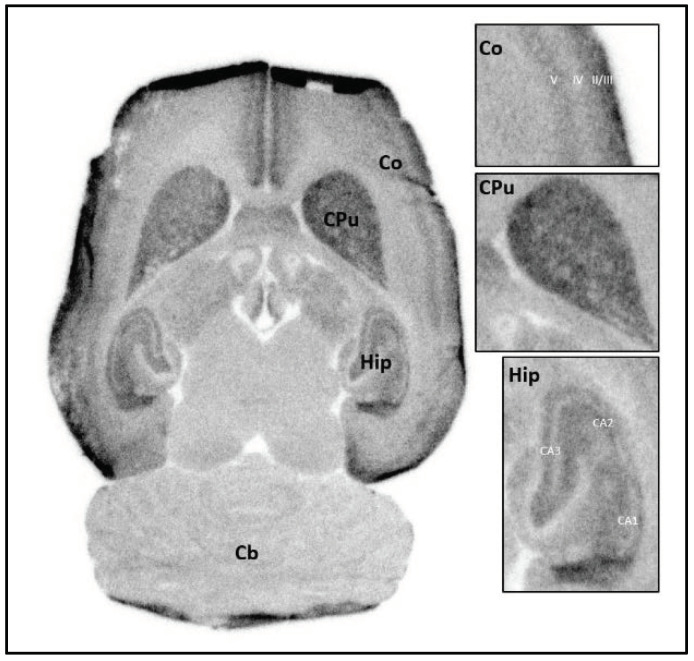
Detailed overview of the binding sites of **[^18^F]11** in rat brain in vitro. Representative autoradiogram of a 12 µm cryosection of one female SPRD rat incubated with 9.5 nM **[^18^F]11**. The regions Co = somatosensory cortex (layers denoted by Roman numerals), CPu = caudate putamen, and Hip = hippocampus (Cornu Ammonis subfields indicated by CA1–3) are presented separately at a higher magnification. Further abbreviations: Cb = cerebellum.

**Figure 2:**
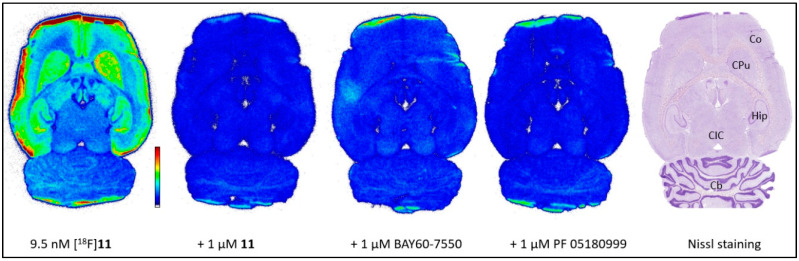
In vitro binding of **[^18^F]11** in rat brain. Representative autoradiograms of 12 µm cryosections of one female SPRD rat incubated with 9.5 nM **[^18^F]11** alone (total binding) or in the presence of 1 µM **11** or the PDE2A-specific compounds BAY60-7550 or PF-0518099. The colour scale corresponds to values of 1001–13754 QL/pixel and is valid for all autoradiograms. The brain regions are labeled in the Nissl stained cryosection: Cb = cerebellum, CIC = colliculi, Co = cortex, CPu = caudate putamen, and Hip = hippocampus.

First, we investigated the binding sites of **[^18^F]11** in rat brains in vitro. Representative autoradiograms, shown in Figure 1 and Figure 2, illustrate the anatomical distribution of **[^18^F]11** in horizontal brain sections obtained from female SPRD and male Wistar rats. High levels of binding are seen in distinct layers of the somatosensory cortex, in different hippocampal regions and in the basal ganglia (Figure 1), the only brain structures with high PDE2A immunoreactivity in rat [41]. For the other brain regions and in particular the cerebellum, all characterized by much lower PDE2A expression [41], much weaker binding of **[^18^F]11** was detectable.

As illustrated in Figure 2, the saturability of the bindings sites of **[^18^F]11** has been proven by co-incubation with an excess of non-labeled **11**. Furthermore, the binding of **[^18^F]11** was completely blocked by the PDE2A-specific inhibitors BAY60-7550 [15] and PF-05180999 [6]. Altogether, these in vitro findings indicate virtually exclusive and high specific binding of **[^18^F]11** towards rat PDE2A.

**Figure 3:**
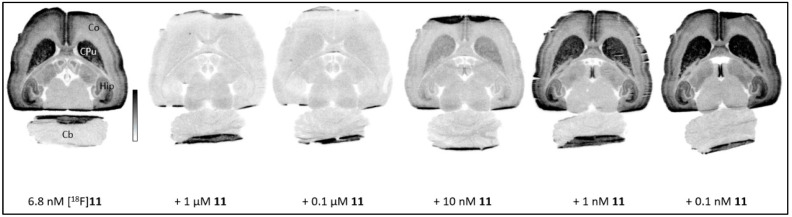
Characterisation of the in vitro binding sites of **[^18^F]11** in rat brain by homologous competition. Representative autoradiograms of 12 µm cryosections of one female SPRD rat incubated with 6.8 nM **[^18^F]11** alone (total binding) or in the presence of different concentrations of **11**. Non-specific binding of the radioligand has been determined by co-incubation with 1 µM BAY60-7550. The grey scale corresponds to values of 1100–11,327 quantum level/pixel and is valid for all autoradiograms. Abbreviations: Cb = cerebellum, Co = cortex, CPu = caudate putamen, and Hip = hippocampus.

**Figure 4:**
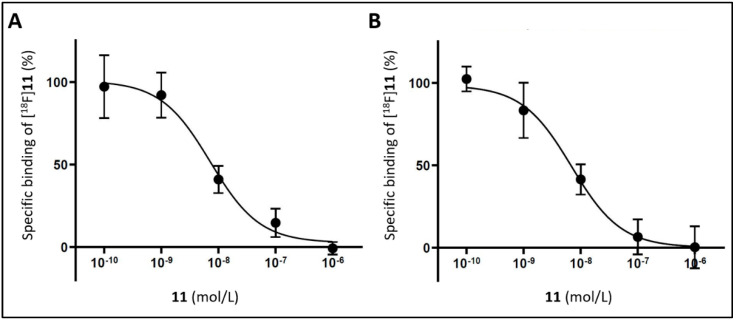
Determination of the *K*_D_ value of the binding of **[^18^F]11** towards rat PDE2A in vitro. Quantitative autoradiography has been performed by incubating 12 µm cryosections of one female SPRD rat (**A**) and one male Wistar-Han rat (**B**) with 6.8 nM **[^18^F]11** alone or with different concentrations of non-labeled **11**. By non-linear analysis of the homologous inhibition curve obtained for the percentage of specific binding of **[^18^F]11** in caudate putamen (GraphPad Prism 9.1.2; Parameters: Nonlinear regression–One site–Homologous), an apparent *K*_D_ value of 0.24 nM (SPRD: 0.38 nM, Wistar: 0.10 nM) and an apparent *B*_max_ value of 16 pmol/mg protein (SPRD: 14 pmol/mg protein, Wistar: 18 pmol/mg protein) has been estimated.

To further characterize the binding site of **[^18^F]11** in PDE2A expressing regions of the rat brain, we performed quantitative in vitro autoradiography with co-incubation of rat brain cryosections with a fixed concentration of **[^18^F]11** supplemented with **11** at different concentrations (Figure 3). The non-linear analyses of the homologous inhibition curves obtained for the caudate nucleus and putamen (Figure 4) resulted in an apparent *K*_D_ value of 0.24 nM and an apparent *B*_max_ value of 16 pmol/mg protein.

Altogether, the autoradiographic studies indicated that **[^18^F]11** binds with high affinity and specificity towards PDE2A in the brain. A binding pattern almost identical to the one reported for the tritiated version of the PDE2A-specific PF-05270430 in rat brain [42] as well as to the immunohistochemically determined distribution of PDE2A in rat [41] along with an affinity and an inhibitory potency towards PDE2A in the low-nanomolar range justified the investigation of the potential of **[^18^F]11** for the imaging of PDE2A in the brain in vivo.

#### 2.3.2. Metabolism of **[^18^F]11** in CD-1 Mice and SPRD Rats

To support the interpretation of the following pharmacokinetic studies, the metabolism of **[^18^F]11** was evaluated in both mice and rats. Non-anesthetized female CD-1 mice (*n* = 2) and one anesthetized female SPRD rat received a single i.v. injection of **[^18^F]11** (~30 MBq) via the tail vein and a blood sample was obtained at 30 min p.i. Immediately afterwards, the animals were sacrificed for sampling of the brains.

**Figure 5:**
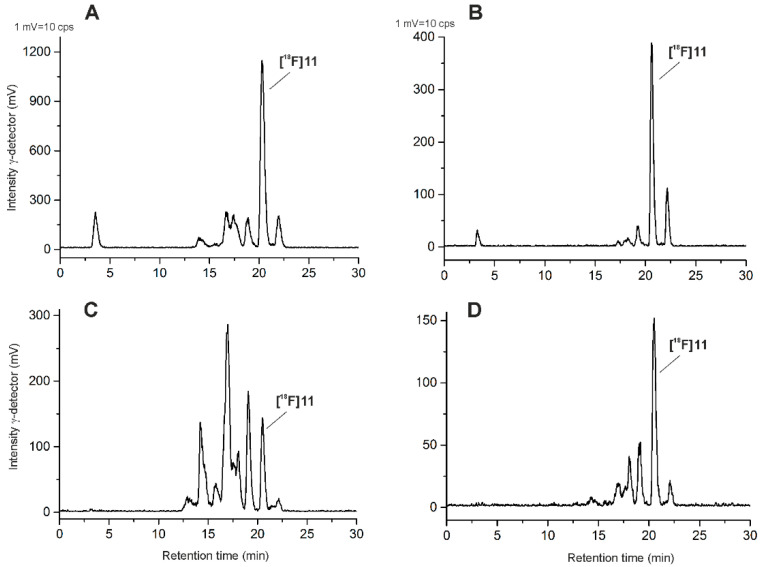
Analytical radio-HPLC chromatograms of extracts of rat plasma (**A**), rat brain (**B**), mouse plasma (**C**) and mouse brain (**D**) samples obtained at 30 min post i.v. injection of **[^18^F]11**.

After deproteinization by treating the plasma and brain samples with a mixture of ACN/H_2_O and extraction of the activity (recoveries ≥ 91%), aliquots were analyzed by radio-HPLC. In rat, the fraction of non-metabolized **[^18^F]11** in the plasma and brain accounted for about 50% and 66%, respectively. In the corresponding radio-chromatograms of the rat plasma samples (Figure 5A) several radiometabolites were detected eluting in front of the radiotracer and thus assumed to be more polar than **[^18^F]11**. One single radiometabolite demonstrates an apparently lipophilic character because it elutes after **[^18^F]11**. For the brain (Figure 5B), a comparable elution pattern is observed in principle, however, with lower intensities of the more polar radiometabolites. Regarding the metabolism of **[^18^F]11** in mouse, no considerable differences could be found in the peak pattern except in the intensities (Figure 5C,D). Thus, in mice the radiotracer is faster degraded resulting in fractions of non-metabolized **[^18^F]11** in plasma and brain accounting for about 7/11% and 42/50% (*n* = 2) of the total activity at 30 min p.i.

#### 2.3.3. Biodistribution and Central Nervous System Penetration Study in CD-1 Mice and SPRD Rats

**Figure 6:**
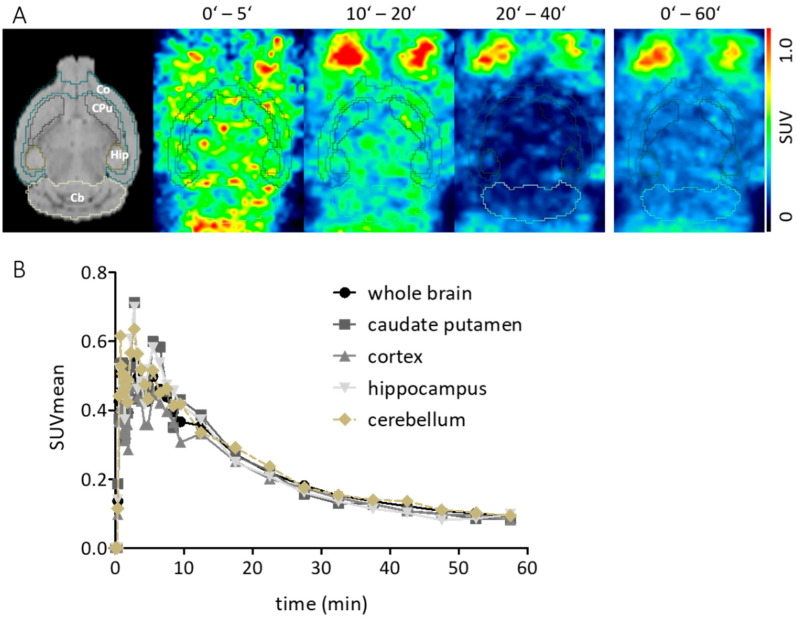
Representative horizontal PET images overlayed with the Ma-benveniste-Mirione-T2 atlas (**A**) and time-activity curves of PDE2A-rich and PDE2A-poor regions of the brain (**B**) of a CD-1 mouse after i.v. injection of **[^18^F]11**. CPu = caudate putamen; Co = cortex; Hip = hippocampus; Cb = cerebellum.

The biodistribution of **[^18^F]11** was further investigated by a dynamic PET scan in one female CD-1 mouse. A moderate brain uptake of **[^18^F]11** is indicated by a peak value of the time-activity curve (TAC) of the brain of SUV = 0.55 at 2.8 min p.i. suggesting a limited passage of the radiotracer through the blood–brain barrier. According to the immunohistological data [41] and the autoradiographic results, the brain regions striatum, cortex and hippocampus were defined as PDE2A-rich regions. However, we observed no significant differences in the uptake of activity in these regions in comparison to the PDE2A-poor reference region cerebellum as illustrated in Figure 6A. Similar TAC peak values (0.71; 0.47; 0.70 at 2.8 min vs. 0.61 at 0.8 min) and washout kinetics indicate poor specificity of **[^18^F]11** in vivo (Figure 6A,B). The high percentage of radiometabolites in the brain (~50% of the activity at 30 min post injection) is probably the main confounding factor hindering the PET imaging with **[^18^F]11**.

Evaluation of the whole-body distribution derived from PET imaging (Figure S14 and Table S1 in Supplementary Materials) revealed a low accumulation of activity in the bladder, spleen, muscle, gallbladder, kidney, and liver (SUV values < 5 at all time points investigated), indicating low renal or hepato-biliary excretion of **[^18^F]11**. An intestinal excretion instead is strongly suggested from the high and continuosly increasing accumulation of activity in the small intestine (maximum at 60 min p.i. with SUV~35). Altogether the biodistribution of activity after i.v. administration does not recapitulate the expression pattern of PDE2A [43].

**Figure 7:**
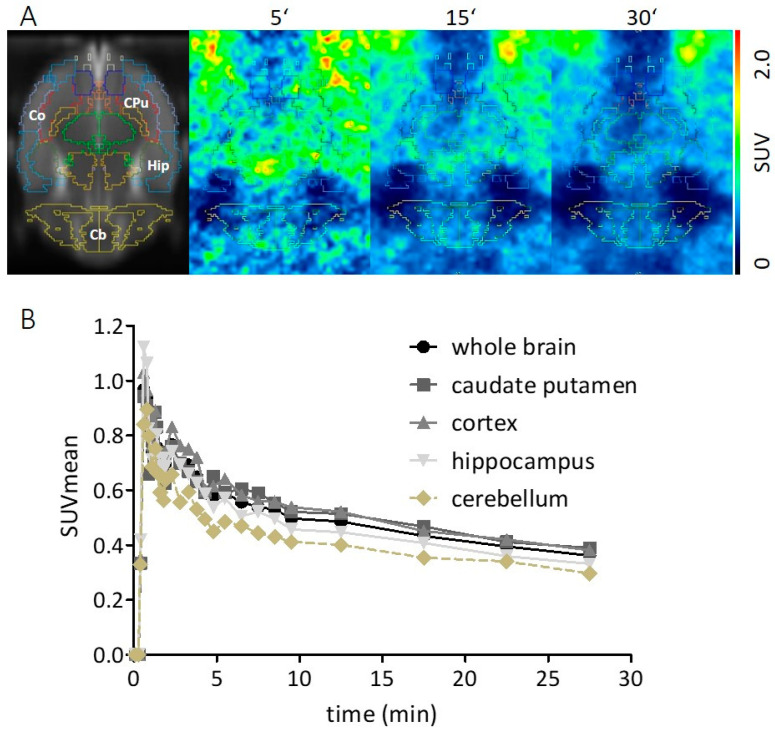
Horizontal PET images of **[^18^F]11** in a rat brain overlayed with the Schwarz-T2 rat atlas (**A**) and time-activity curves of PDE2A-rich and PDE2A-poor regions of the brain (**B**) of an SPRD rat after i.v. injection of **[^18^F]11** (*n* = 1, SUVmean). Cb = cerebellum; Co = cortex; Cpu = caudate putamen; Hip = hippocampus.

To assess potential species differences in the pharmacokinetics of **[^18^F]11**, the tracer was administered intravenously in one female SPRD rat for investigation by dynamic PET. Figure 7A illustrates a higher but still moderate brain uptake of **[^18^F]11** in the rat in comparison to the mouse confirmed by a peak value of 0.97 at 0.6 min (Figure 7B). The PDE2A-rich regions caudate nucleus and putamen, cortex and hippocampus present a slightly higher uptake over time than the PDE2A-poor region cerebellum (signal-to-background ratio between 1.1 and 1.4) insufficient for imaging purposes as observed on the PET images (Figure 7A). The non-negligible fraction of 34% brain penetrant radiometabolites at 30 min p.i. probably contributes to high background noise, hindering the detection of a potential specific binding of **[^18^F]11** in vivo. Furthermore, the signal-to-background ratio in vivo under dynamic conditions might per se be lower than anticipated from the autoradiographic experiments performed under equilibrium conditions in vitro (caudate putamen-to-cerebellum ratio of about 2) [44].

These results demonstrated (i) an average brain uptake of **[^18^F]11** in mice and rats with (ii) no significant uptake in PDE2A-expressing regions and (iii) a contribution of radiometabolites to the measured PET signals due to the passage of a non-negligible fraction of radiometabolites through the blood–brain barrier in both species despite a slightly slower metabolization in rat in comparison to mouse.

Altogether, regardless of the limited success to confirm the excellent in vitro potential of **[^18^F]11** to detect PDE2A protein in the brain in vivo, we conclude that **[^18^F]11** has potential as a starting point for further structural optimization to develop a PDE2A-specific PET ligand for neuroimaging of this enzyme in vivo.

## 3. Materials and Methods

### 3.1. Organic Chemistry

#### 3.1.1. General

Commercially available reagents and solvents were used as received. Reactions requiring anhydrous conditions were conducted in an inert atmosphere using argon and the Schlenk technique with dried reagents and solvents. Ethyl 2-amino-5-butyrylbenzoate **14** [28] and 3-chloro-6-cyclopropyl-2-methylpyrido[2,3-*b*]pyrazine [20] were prepared according to literature procedures.

For thin-layer chromatography (TLC), aluminum sheets coated with silica gel 60, F_254_ (Merck, Darmstadt, Germany) were used. Analytes were detected by irradiation with a UV lamp (254 nm). Flash chromatography was accomplished manually on silica gel 60, 40–63 µm, 230–400 mesh (VWR International, Darmstadt, Germany) or using a Teledyne Isco Combi Flash NEXTGEN 300+ with RediSep^®^ Rf cartridges prepacked with normal phase silica (35 to 70 µm).

For HPLC analysis, purified water from a Simplicity 185 water purification system (Millipore, Merck) with addition of 0.1% trifluoroacetic acid (TFA, component A) and acetonitrile (component B) ≥ 99.9 %, HiPerSolv CHROMANORM^®^ (VWR) were used as eluents. Analytical HPLC was performed on a Dionex UltiMate 3000 (Thermo Fisher Scientific, Schwerte, Germany) system using a NUCLEODUR 100-5 C18 ec column (5 µm, 250 × 4.6 mm from Macherey-Nagel, Düren, Germany) thermostated at 25 °C, with a flow rate of 1 mL/min and UV detection at 230 nm. The following method was used for the separations: t [min]/B [%]: 0/5, 5/5, 15/95, 25/95, 26/5, 31/5. Preparative HPLC was performed on a Varian ProStar system with a NUCLEODUR C18 HTec column (5 µm, 150 × 32 mm) at a flow rate of 15 mL/min and UV detection at 230 nm.

NMR spectra were recorded at room temperature on a Varian MERCURYplus 300 (^1^H: 300 MHz, ^13^C: 75 MHz, ^19^F: 282 MHz), a Varian MERCURYplus 400 (^1^H: 400 MHz, ^13^C: 100 MHz, ^19^F: 376 MHz) or a Bruker AVANCE III HD 400 (^1^H: 400 MHz, ^13^C: 100 MHz, ^19^F: 376 MHz). The signal of the residual solvent (CDCl_3_: 7.26 ppm (^1^H), 77.16 ppm (^13^C) and DMSO-d_6_: 2.50 ppm (^1^H), 39.52 ppm (^13^C)) was used for referencing tetramethylsilane. Multiplicities (m—multiplet, s—singlet, d—doublet, t—triplet, q—quartet and combination thereof) were reported as observed and might differ from the expected signal multiplicity. Carbon-13 spectra were proton-decoupled and measured as APT spectra. Diastereomeric ratios (*dr*) were estimated using ^1^H or ^19^F spectra.

High resolution mass spectra were recorded with an ESI-TOF micrOTOF (Bruker Daltonik GmbH, Bremen, Germany) mass spectrometer linked to an Agilent 1100 HPLC (Agilent Technologies GmbH, Waldbronn, Germany) controlled by otofControl 3.4 and HyStar 3.2-LC/MS or with an ESI-qTOF Impact II (Bruker Daltonik GmbH) mass spectrometer linked to a Dionex Ultimate 3000 UHPLC (Thermo Fisher Scientific) controlled by otofControl 4.0 and HyStar 3.2-LC/MS.

#### 3.1.2. Syntheses

##### General Procedure 1 (GP1)

An 0.33 M solution of ethyl 2-halo-5-iodobenzoate in dry THF was cooled to −40 to −50 °C and the temperature was maintained during the whole procedure. 1.1 eq of 1.3 M *i*PrMgCl · LiCl in THF were added during 15 to 20 min. After 1 h stirring, 1.2 eq of freshly distilled butanal was added over 10 to 15 min and the mixture was stirred for an additional hour. An aqueous saturated NH_4_Cl solution (20 mL) was added and the cooling was removed. After the solution was warmed to room temperature, 100 mL DCM was added and the aqueous phase was extracted three times with 20 mL DCM. The combined organic phases were dried over MgSO_4_, filtered and the solvent was removed under reduced pressure. The crude product was purified by flash chromatography.

##### Ethyl 2-fluoro-5-(1-hydroxybutyl)benzoate **3**

Compound **3** was prepared according to GP1 from 2.95 g (10.0 mmol) ethyl 2-fluoro-5-iodobenzoate, 30 mL THF, 8.5 mL (11.0 mmol) 1.3 M *i*PrMgCl · LiCl in THF and 1.1 mL (12.0 mmol) butanal, using a gradient from 0 to 40% ethyl acetate in cyclohexane for flash chromatography and yielding 1.16 g (48%) of the benzyl alcohol as a colorless oil.

**^1^H NMR** (400 MHz, CDCl_3_): δ = 7.85 (dd, *J* = 6.9, 2.4 Hz, 1H), 7.48 (ddd, *J* = 8.6, 4.6, 2.4 Hz, 1H), 7.08 (dd, *J* = 10.5, 8.5 Hz, 1H), 4.69 (dd, *J* = 7.6, 5.6 Hz, 1H), 4.38 (q, *J* = 7.1 Hz, 2H), 2.13 (s, 1H), 1.70 (m, 2H), 1.38 (t, *J* = 7.2 Hz, 4H), 1.35 (m, 1H), 0.92 (t, *J* = 7.4 Hz, 3H) ppm. **^13^C NMR** (101 MHz, CDCl_3_): δ = 164.6 (d, *J* = 3.7 Hz), 161.2 (d, *J* = 259.2 Hz), 140.9 (d, *J* = 3.8 Hz), 131.9 (d, *J* = 9.0 Hz), 129.5, 118.8 (d, *J* = 10.2 Hz), 117.1 (d, *J* = 22.7 Hz), 73.4, 61.5, 41.4, 19.0, 14.4, 14.0 ppm. **^19^F NMR** (377 MHz, CDCl_3_): δ = −111.9 (m) ppm. **HR-MS** (ESI) *m*/*z* calcd. for C_13_H_18_FO_3_^+^: 241.1234, found: 241.1239.

##### Ethyl 2-bromo-5-(1-hydroxybutyl)benzoate **4**

Compound **4** was prepared according to GP1 from 7.10 g (20.0 mmol) ethyl 2-bromo-5-iodobenzoate, 60 mL THF, 16.9 mL (22.0 mmol) 1.3 M *i*PrMgCl · LiCl in THF and 2.2 mL (24.0 mmol) butanal, using a gradient from 0 to 30% ethyl acetate in cyclohexane for flash chromatography and yielding 5.09 g (84%) of the benzyl alcohol as a colorless oil.

**^1^H NMR** (400 MHz, CDCl_3_): δ = 7.72 (d, *J* = 2.3 Hz, 1H), 7.60 (d, *J* = 8.2 Hz, 1H), 7.29 (dd, *J* = 8.3, 2.3 Hz, 1H), 4.68 (t, *J* = 6.7 Hz, 1H), 4.39 (q, *J* = 7.1 Hz, 2H), 2.06 (s, 1H), 1.68 (m, 2H), 1.40 (t, *J* = 7.2 Hz, 3H), 1.36 (m, 2H), 0.92 (t, *J* = 7.4 Hz, 3H) ppm. **^13^C NMR** (101 MHz, CDCl_3_): δ = 166.5, 144.6, 134.4, 132.6, 130.0, 128.7, 120.2, 73.4, 61.9, 41.3, 19.0, 14.3, 14.0 ppm. **HR-MS** (ESI) *m*/*z* calcd. for C_13_H_17_BrNaO_3_^+^: 323.0253, found: 323.0242.

##### General Procedure 2 (GP2)

A 0.33 M solution of the benzyl alcohol in dry DCM was cooled in an ice bath. Subsequently 1 mol% TsOH · H_2_O and 1.2 eq 3,4-dihydro-2*H*-pyran were added. After stirring for 16 h at room temperature, 10 mL of an aqueous saturated NaHCO_3_ solution was added and the mixture was stirred for 1 h and then extracted three times with 20 mL DCM. The combined organic phases were dried over Na_2_SO_4_, filtered and the solvent was removed at reduced pressure. The crude product was purified by flash chromatography.

##### Ethyl 2-fluoro-5-(1-[(tetrahydro-2*H*-pyran-2-yl)oxy]butyl)benzoate **5**

Compound **5** was prepared according to GP2 from 401 mg (1.67 mmol) **3**, 5 mL DCM, 3.2 mg (0.0167 mmol) TsOH · H_2_O, 183 µL (2.00 mmol) 3,4-dihydro-2*H*-pyran, using 5% ethyl acetate in cyclohexane for flash chromatography and yielding 468 mg (87%) of the diastereomeric mixture of the protected alcohol as a colorless oil.

**^1^H NMR** (400 MHz, CDCl_3_): δ = 7.87 (dd, *J* = 7.0, 2.4 Hz, 0.2H), 7.82 (dd, *J* = 7.0, 2.3 Hz, 0.8H), 7.52 (ddd, *J* = 8.5, 4.6, 2.4 Hz, 0.2H), 7.44 (ddd, *J* = 8.5, 4.6, 2.4 Hz, 0.8H), 7.08 (m, 1H), 4.79 (t, *J* = 3.4 Hz, 0.2H), 4.69 (dd, *J* = 7.9, 5.7 Hz, 0.8H), 4.56 (t, *J* = 6.5 Hz, 0.2H), 4.38 (q, *J* = 7.1 Hz, 2H), 4.34 (t, *J* = 3.5 Hz, 0.8H), 3.92 (m, 0.8H), 3.49 (m, 1H), 3.26 (m, 0.2H), 1.83 (m, 2H), 1.56 (m, 7H), 1.39 (t, *J* = 7.1 Hz, 3H), 1.29 (m, 1H), 0.90 (m, 3H) ppm. **^13^C NMR** (101 MHz, CDCl_3_): δ = 164.6 (d, *J* = 3.5 Hz), 161.3 (d, *J* = 259.3 Hz), 161.1 (d, *J* = 258.8 Hz), 139.9 (d, *J* = 4.3 Hz), 138.8 (d, *J* = 3.6 Hz), 132.8 (d, *J* = 9.0 Hz), 132.5 (d, *J* = 9.0 Hz), 130.6, 130.0, 118.9 (d, *J* = 10.1 Hz), 118.5 (d, *J* = 10.1 Hz), 117.1 (d, *J* = 22.8 Hz), 116.8 (d, *J* = 22.8 Hz), 98.6, 95.3, 78.2, 75.9, 62.4, 62.3, 61.5, 61.4, 40.5, 39.5, 30.8, 25.6, 25.5, 19.5, 19.4, 19.3, 18.7, 14.4, 14.1, 14.1 ppm. **^19^F NMR** (376 MHz, CDCl3): δ = −111.9 (m, 0.8H), −112.6 (m, 0.2H) ppm. ***dr*** (^19^F NMR): 0.8/0.2. **HR-MS** (ESI) *m*/*z* calcd. for C_18_H_25_FNaO_4_^+^: 347.1629, found: 347.1641.

##### Ethyl 2-bromo-5-(1-[(tetrahydro-2H-pyran-2-yl)oxy]butyl)benzoate **6**

Compound **6** was prepared according to GP2 from 2.51 g (8.34 mmol) **4**, 25 mL DCM, 15.9 mg (0.0834 mmol) TsOH · H_2_O, 913 µL (10.0 mmol) 3,4-dihydro-2*H*-pyran, using a gradient from 0 to 10% ethyl acetate in cyclohexane for flash chromatography and yielding 2.96 g (92%) of the diastereomeric mixture of the protected alcohol as a colorless oil.

**^1^H NMR** (400 MHz, CDCl_3_): δ = 7.74 (d, *J* = 2.2 Hz, 0.2H), 7.67 (d, *J* = 2.2 Hz, 0.8H), 7.59 (m, 1H), 7.34 (dd, *J* = 8.2, 2.2 Hz, 0.2H), 7.26 (dd, *J* = 8.2, 2.2 Hz, 0.8H), 4.80 (t, *J* = 3.4 Hz, 0.2H), 4.68 (dd, *J* = 7.9, 5.6 Hz, 0.8H), 4.55 (t, *J* = 6.5 Hz, 0.2H), 4.40 (q, *J* = 7.1 Hz, 2H), 4.35 (t, *J* = 3.5 Hz, 0.8H), 3.92 (m, 0.8H), 3.49 (m, 1H), 3.27 (m, 0.2H), 1.82 (m, 2H), 1.55 (m, 7H), 1.41 (t, *J* = 7.1 Hz, 3H), 1.30 (m, 1H), 0.90 (m, 3H) ppm. **^13^C NMR** (75 MHz, CDCl_3_): δ = 166.4, 143.5, 142.5, 134.4, 134.1, 132.7, 132.2, 131.0, 130.7, 129.8, 129.4, 120.3, 119.8, 98.6, 95.5, 78.0, 76.0, 62.5, 62.2, 61.8, 61.7, 40.4, 39.4, 30.8, 25.6, 25.5, 19.5, 19.3, 19.3, 18.6, 14.3, 14.1, 14.1 ppm. ***dr*** (^1^H NMR): 0.8/0.2. **HR-MS** (ESI) *m*/*z* calcd. for C_18_H_25_BrNaO_4_^+^: 407.0828, found: 407.0832.

##### General Procedure 3 (GP3)

To a solution of the ester in methanol, hydrazine hydrate was added and the mixture was stirred at reflux or at room temperature for the given time. Water was added and the aqueous phase was extracted with DCM. The combined organic phases were dried over Na_2_SO_4_, filtered and the solvent was removed at reduced pressure.

##### 2-Fluoro-5-(1-[(tetrahydro-2H-pyran-2-yl)oxy]butyl)benzohydrazide **7**

Compound **7** was prepared according to GP3 from 526 mg (1.0 eq, 1.62 mmol) ester **5**, 7.0 mL methanol and 787 µL (10.0 eq, 16.2 mmol) hydrazine hydrate. The solution was heated to reflux for 4 h, cooled to room temperature and 20 mL water and 20 mL DCM were added. The mixture was extracted three times with 20 mL DCM. The crude product was purified by flash chromatography using a gradient from 0 to 5% methanol in DCM yielding 309 mg (61%) of the diastereomeric mixture of the hydrazide as a viscous colorless oil.

**^1^H NMR** (300 MHz, CDCl_3_): δ = 7.99 (m, 2H), 7.52 (ddd, *J* = 8.5, 5.1, 2.4 Hz, 0.25H), 7.42 (ddd, *J* = 8.5, 5.0, 2.4 Hz, 0.75H), 7.08 (m, 1H), 4.78 (t, *J* = 3.4 Hz, 0.25H), 4.72 (dd, *J* = 7.8, 5.7 Hz, 0.75H), 4.59 (t, *J* = 6.5 Hz, 0.25H), 4.32 (t, *J* = 3.5 Hz, 0.75H), 4.17 (s, 2H), 3.91 (m, 0.75H), 3.48 (m, 1H), 3.26 (m, 0.25H), 1.60 (m, 10H), 0.89 (m, 3H) ppm. **^13^C NMR** (75 MHz, CDCl_3_): δ = 164.7, 164.6, 161.6, 158.3, 140.0, 139.9, 132.1, 132.0, 131.9, 131.8, 130.4, 130.4, 129.8, 129.8, 119.2, 119.0, 116.4, 116.1, 115.8, 98.7, 95.5, 78.2, 76.0, 62.5, 62.4, 40.5, 39.4, 30.8, 25.6, 25.5, 19.6, 19.5, 19.3, 18.7, 14.1, 14.1 ppm. **^19^F NMR** (282 MHz, CDCl_3_): δ = −114.3 (m, 0.75F), −115.0 (m, 0.25F) ppm. ***dr*** (^19^F NMR): 0.75/0.25. **HR-MS** (ESI) *m*/*z* calcd. for C_16_H_23_FN_2_NaO_3_^+^: 333.1585, found: 333.1584.

##### 2-Bromo-5-(1-[(tetrahydro-2H-pyran-2-yl)oxy]butyl)benzohydrazide **8**

Compound **8** was prepared according to GP3 from 2.21 g (1.0 eq, 5.73 mmol) ester **6**, 10 mL methanol and 2.8 mL (10.0 eq, 57.3 mmol) hydrazine hydrate. The solution was heated to reflux for 5.5 h, cooled to room temperature and 20 mL water and 20 mL DCM were added. The mixture was extracted three times with 20 mL DCM. The crude product was purified by flash chromatography using a gradient from 0 to 5% methanol in DCM yielding 1.88 g (89%) of the diastereomeric mixture of the hydrazide as a viscous colorless oil.

**^1^H NMR** (400 MHz, CDCl_3_): δ = 7.54 (m, 1H), 7.47 (d, *J* = 2.2 Hz, 0.2H), 7.41 (d, *J* = 2.2 Hz, 0.8H), 7.35 (d, *J* = 21.3 Hz, 1H), 7.29 (dd, *J* = 8.3, 2.2 Hz, 0.2H), 7.23 (dd, *J* = 8.2, 2.2 Hz, 0.8H), 4.75 (dd, *J* = 4.2, 2.9 Hz, 0.2H), 4.65 (dd, *J* = 7.9, 5.6 Hz, 0.8H), 4.53 (t, *J* = 6.4 Hz, 0.2H), 4.33 (t, *J* = 3.4 Hz, 0.8H), 4.13 (d, *J* = 3.7 Hz, 2H), 3.89 (ddd, *J* = 11.6, 7.6, 4.5 Hz, 0.8H), 3.48 (m, 1H), 3.27 (m, 0.2H), 1.78 (m, 3H), 1.53 (m, 6H), 1.28 (m, 1H), 0.89 (m, 3H) ppm. **^13^C NMR** (101 MHz, CDCl_3_): δ = 168.8, 168.7, 143.9, 143.0, 135.9, 135.5, 133.6, 133.3, 130.3, 130.0, 128.3, 127.8, 118.4, 117.9, 98.8, 95.4, 78.1, 75.9, 62.6, 62.4, 40.4, 39.3, 30.8, 30.8, 25.6, 25.4, 19.6, 19.4, 19.3, 18.6, 14.1, 14.1 ppm. ***dr*** (^1^H NMR): 0.8/0.2. **HR-MS** (ESI) *m*/*z* calcd. for C_16_H_23_BrN_2_NaO_3_^+^: 393.0784, found: 393.0785.

##### General Procedure 4 (GP4)

To a 0.1 M solution of 1.0 eq hydrazide in ACN were added 6.0 eq DBU and 3-chloro-6-cyclopropyl-2-methylpyrido[2,3-*b*]pyrazine. After stirring at room temperature for 4 h, 2.0 eq PPh_3_ was added. 15 min later, the solution was placed in an ice bath and 2.0 eq C_2_Cl_6_ was added portionwise. The mixture was stirred for an additional hour at room temperature, silica gel was added and evaporated to dryness at reduced pressure. The residue was purified by flash chromatography.

##### 2-Cyclopropyl-9-(2-fluoro-5-(1-[(tetrahydro-2H-pyran-2-yl)oxy]butyl)phenyl)-6-methylpyrido[3,2-e][1,2,4]triazolo[4,3-a]pyrazine **9**

Compound **9** was prepared according to GP4 from 278 mg (0.897 mmol) **7**, 9.0 mL ACN, 805 µL (5.38 mmol) DBU, 197 mg (1.0 eq, 0.897 mmol) 3-chloro-6-cyclopropyl-2-methylpyrido[2,3-*b*]pyrazine, 471 mg (1.79 mmol) PPh_3_ and 425 mg (1.79 mmol) C_2_Cl_6_, using isocratic conditions at 50% ethyl acetate in cyclohexane for flash chromatography and yielding 245 mg (57 %) of the diastereomeric mixture of the cyclized product as a light yellow solid.

**TLC**: R_f_ = 0.50 (MeOH/DCM = 5/95). **HPLC**: *t*_R_ = 20.97 min (95.0 %). **^1^H NMR** (400 MHz, CDCl_3_): δ = 8.14 (m, 1H), 7.59 (m, 2H), 7.46 (m, 1H), 7.20 (m, 1H), 4.85 (t, *J* = 3.3 Hz, 0.4H), 4.77 (dd, *J* = 7.7, 5.9 Hz, 0.6H), 4.66 (t, *J* = 6.4 Hz, 0.4H), 4.48 (dd, *J* = 4.3, 3.0 Hz, 0.6H), 3.94 (m, 0.6H), 3.59 (ddd, *J* = 12.2, 9.4, 3.1 Hz, 0.4H), 3.50 (dt, *J* = 10.7, 4.3 Hz, 0.6H), 3.32 (m, 0.4H), 3.04 (s, 3H), 1.96 (m, 1H), 1.85 (m, 2H), 1.69 (m, 3H), 1.53 (m, 4H), 1.37 (m, 1H), 0.92 (m, 3H), 0.82 (m, 2H), 0.51 (m, 2H) ppm. **^13^C NMR** (101 MHz, CDCl_3_): δ = 163.5, 163.5, 162.0, 161.6, 159.5, 159.2, 152.2, 146.8, 145.5, 145.2, 139.9, 139.9, 138.8, 138.8, 137.8, 137.8, 137.0, 137.0, 130.8, 130.8, 130.5, 130.4, 130.4, 130.3, 129.9, 129.9, 129.2, 122.8, 122.8, 117.8, 117.7, 117.4, 117.2, 115.7, 115.5, 115.4, 115.2, 98.1, 95.5, 77.8, 76.2, 62.6, 62.1, 40.6, 39.6, 30.9, 30.8, 29.8, 25.6, 25.6, 21.2, 19.6, 19.3, 19.2, 18.7, 17.5, 17.4, 14.2, 14.1, 11.8, 11.8, 11.7, 11.3 ppm. **^19^F NMR** (377 MHz, CDCl_3_): δ = −111.3 (m, 0.6F), −111.7 (m, 0.4F) ppm. ***dr*** (^19^F NMR): 0.6/0.4. **HR-MS** (ESI) *m*/*z* calcd. for C_27_H_31_FN_5_O_2_^+^: 476.2456, found: 476.2456.

##### 9-(2-Bromo-5-(1-[(tetrahydro-2H-pyran-2-yl)oxy]butyl)phenyl)-2-cyclopropyl-6-methylpyrido[3,2-e][1,2,4]triazolo[4,3-a]pyrazine **10**

Compound **10** was prepared according to GP4 from 371 mg (1.00 mmol) **8**, 10.0 mL ACN, 897 µL (6.00 mmol) DBU, 220 mg (1.0 eq, 1.00 mmol) 3-chloro-6-cyclopropyl-2-methylpyrido[2,3-*b*]pyrazine, 471 mg (1.79 mmol) PPh_3_ and 425 mg (1.79 mmol) C_2_Cl_6_, using a gradient from 0 to 75% ethyl acetate in cyclohexane for flash chromatography and yielding 381 mg (71%) of the diastereomeric mixture of the cyclized product as a white solid.

**^1^H NMR** (400 MHz, CDCl_3_): δ = 8.14 (m, 1H), 7.69 (m, 1H), 7.45 (m, 3H), 4.83 (d, *J* = 13.0 Hz, 0.2H), 4.74 (t, *J* = 6.7 Hz, 0.8H), 4.64 (t, *J* = 6.4 Hz, 0.2H), 4.49 (d, *J* = 13.5 Hz, 0.8H), 3.91 (d, *J* = 10.7 Hz, 0.8H), 3.60 (m, 0.2H), 3.46 (m, 0.8H), 3.33 (m, 0.2H), 3.05 (s, 3H), 1.89 (m, 3H), 1.62 (m, 8H), 0.90 (m, 4H), 0.68 (m, 2H), 0.16 (m, 1H) ppm. **^13^C NMR** (101 MHz, CDCl_3_): δ = 163.5, 152.1, 148.8, 148.6, 146.3, 143.3, 142.4, 137.4, 136.9, 136.9, 132.5, 132.1, 131.2, 130.8, 130.3, 130.1, 129.7, 129.0, 124.4, 122.8, 122.7, 95.9, 95.5, 76.3, 76.1, 62.8, 62.4, 40.6, 39.5, 30.9, 30.9, 30.8, 30.7, 25.6, 21.2, 19.7, 19.5, 19.3, 18.5, 17.3, 17.2, 14.2, 14.1, 12.6, 11.3, 10.9 ppm. ***dr*** (^1^H NMR): 0.8/0.2. **HR-MS** (ESI) *m*/*z* calcd. for C_27_H_31_BrN_5_O_2_^+^: 536.1656, found: 536.1647.

##### 1-[3-(2-Cyclopropyl-6-methylpyrido[3,2-e][1,2,4]triazolo[4,3-a]pyrazin-9-yl)-4-fluorophenyl]butan-1-ol **11**

To a solution of 139 mg (1.0 eq, 0.292 mmol) **9** in 5.8 mL methanol were added 7.3 mg (10 mol%, 0.0292 mmol) pyridinium *p*-toluenesulfonate and the mixture was stirred for seven days at room temperature. Water (100 mL) was added and the mixture was extracted five times with 20 mL DCM. The combined organic phases were dried over Na_2_SO_4_, filtered and the solvent was removed at reduced pressure. The crude product was purified by flash chromatography using a gradient from 0 to 100% ethyl acetate in cyclohexane yielding 89 mg (78%) of the benzyl alcohol as a light yellow solid.

**TLC**: R_f_ = 0.26 (MeOH/DCM = 5/95). **HPLC**: *t*_R_ = 16.46 min (>99.2 %). **^1^H NMR** (400 MHz, CDCl_3_): δ = 8.15 (d, *J* = 8.3 Hz, 1H), 7.64 (dd, *J* = 6.6, 2.3 Hz, 1H), 7.60 (ddd, *J* = 8.5, 5.0, 2.3 Hz, 1H), 7.46 (d, *J* = 8.3 Hz, 1H), 7.21 (m, 1H), 4.79 (dd, *J* = 7.4, 5.8 Hz, 1H), 3.04 (s, 3H), 2.30 (br s, 1H), 1.96 (tt, *J* = 8.0, 4.6 Hz, 1H), 1.78 (m, 2H), 1.41 (m, 2H), 0.93 (t, *J* = 7.3 Hz, 3H), 0.83 (m, 2H), 0.50 (br s, 2H) ppm. **^13^C NMR** (75 MHz, CDCl_3_): δ = 163.6, 160.6 (d, *J* = 249.6 Hz), 152.2, 146.7, 145.3, 141.1 (d, *J* = 3.5 Hz), 137.7, 136.9, 129.8 (d, *J* = 8.3 Hz), 129.5 (d, *J* = 2.4 Hz), 129.0, 122.9, 117.5 (d, *J* = 15.2 Hz), 115.7 (d, *J* = 21.0 Hz), 73.5, 41.5, 21.1, 19.0, 17.5, 14.1, 11.9, 11.8 ppm. **^19^F NMR** (282 MHz, CDCl_3_): δ = −111.1 (m) ppm. **HR-MS** (ESI) *m*/*z* calcd. for C_22_H_23_FN_5_O^+^: 392.1881, found: 392.1886.

##### 1-[3-(2-Cyclopropyl-6-methylpyrido[3,2-e][1,2,4]triazolo[4,3-a]pyrazin-9-yl)-4-fluorophenyl]butan-1-one **12**

To a solution of 35.7 mg (1.0 eq, 0.0912 mmol) benzyl alcohol **11** in 3.6 mL DCM were added 46.4 mg (1.2 eq, 0.109 mmol) Dess–Martin periodinane at 0 °C. After the addition, the cooling bath was removed and the reaction mixture was stirred for 2 h at room temperature. Water (20 mL) was added and the mixture was extracted with 50 mL DCM. The organic phase was separated, dried over Na_2_SO_4_, filtered and the solvent was removed at reduced pressure. Flash chromatography using 50% ethyl acetate in cyclohexane yielded 35 mg (99%) of the butyrophenone as a white solid.

**TLC**: R_f_ = 0.44 (MeOH/DCM = 5/95). **HPLC**: *t*_R_ = 17.33 min (99.6 %). **^1^H NMR** (400 MHz, CDCl_3_): δ = 8.27 (m, 2H), 8.17 (d, *J* = 8.3 Hz, 1H), 7.47 (d, *J* = 8.3 Hz, 1H), 7.34 (t, *J* = 8.7 Hz, 1H), 3.05 (s, 3H), 2.98 (t, *J* = 7.3 Hz, 2H), 1.97 (tt, *J* = 8.1, 4.6 Hz, 1H), 1.79 (m, 2H), 1.00 (t, *J* = 7.4 Hz, 3H), 0.85 (dd, *J* = 8.0, 3.0 Hz, 2H), 0.46 (m, 2H) ppm. **^13^C NMR** (101 MHz, CDCl_3_): δ = 198.3, 164.0 (d, *J* = 258.3 Hz), 163.7, 152.1, 146.9, 144.3, 137.6, 137.2, 133.4 (d, *J* = 3.3 Hz), 132.7 (d, *J* = 3.4 Hz), 132.6 (d, *J* = 9.5 Hz), 129.2, 123.0, 118.2 (d, *J* = 15.7 Hz), 116.2 (d, *J* = 21.4 Hz), 40.7, 21.2, 17.8, 17.5, 14.0, 11.9 ppm. **^19^F NMR** (377 MHz, CDCl_3_): δ = −101.5 (m) ppm. **HRMS** (ESI) *m*/*z* calcd. for C_22_H_21_FN_5_O^+^: 390.1725, found: 390.1730.

##### 2-Cyclopropyl-6-methyl-9-(5-(1-[(tetrahydro-2H-pyran-2-yl)oxy]butyl)-2-(4,4,5,5-tetramethyl-1,3,2-dioxaborolan-2-yl)phenyl)pyrido[3,2-e][1,2,4]triazolo[4,3-a]pyrazine **13**

A mixture of 268 mg (1.0 eq, 0.500 mmol) **10**, 140 mg (1.1 eq, 0.550 mmol) B_2_pin_2_, 18.3 mg (5 mol%, 0.025 mmol) [Pd(dppf)Cl_2_] and 147 mg (3.0 eq, 1.50 mmol) KOAc in 3 mL dry dioxane was heated for 72 h at 80 °C. Additional 140 mg (1.1 eq, 0.550 mmol) B_2_pin_2_ and 18.3 mg (5 mol%, 0.025 mmol) [Pd(dppf)Cl_2_] were added. After a further 22 h at 80 °C, 10 mL water was added and the mixture was extracted three times with 20 mL DCM. The combined organic phases were dried over Na_2_SO_4_, filtered and the solvent was removed at reduced pressure. The crude product was purified by flash chromatography using a gradient from 0 to 100% ethyl acetate in cyclohexane. Further purification with preparative HPLC at isocratic conditions using 80% ACN in water yielded 67 mg (23%) of the diastereomeric mixture of the boronic acid pinacol ester as a beige solid.

**TLC**: R_f_ = 0.40 (MeOH/DCM = 5/95). **HPLC**: *t*_R_ = 23.52 min (94.0%). **^1^H NMR** (400 MHz, CDCl_3_): δ = 8.09 (m, 1H), 7.97 (m, 1H), 7.59 (m, 0.4H), 7.52 (m, 1.6H), 7.37 (m, 1H), 4.87 (t, *J* = 3.2 Hz, 0.2H), 4.80 (dd, *J* = 7.7, 5.8 Hz, 0.8H), 4.69 (t, *J* = 6.3 Hz, 0.2H), 4.49 (dd, *J* = 4.5, 3.0 Hz, 0.8H), 3.94 (m, 0.8H), 3.61 (m, 0.2H), 3.48 (m, 0.8H), 3.32 (m, 0.2H), 3.05 (s, 3H), 1.86 (m, 3H), 1.61 (m, 7H), 1.37 (m, 1H), 0.90 (m, 4H), 0.71 (m, 13H), 0.39 (m, 2H) ppm. **^13^C NMR** (101 MHz, CDCl_3_): δ = 163.1, 163.0, 152.2, 151.6, 151.3, 146.7, 145.9, 145.8, 138.5, 138.4, 136.8, 135.6, 135.3, 135.0, 134.6, 128.8, 128.8, 128.3, 127.7, 127.2, 122.2, 122.2, 97.8, 95.9, 83.3, 83.3, 78.0, 76.9, 62.7, 61.9, 40.5, 39.5, 31.0, 30.7, 25.7, 25.6, 24.4, 21.2, 19.8, 19.3, 19.1, 18.6, 17.4, 17.3, 14.2, 14.1, 11.8, 11.7 ppm. **^11^B NMR** (128 MHz, CDCl_3_): δ = 30.8 ppm. ***dr*** (^1^H NMR): 0.8/0.2. **HR-MS** (ESI) *m*/*z* calcd. for C_33_H_42_BN_5_NaO_4_^+^: 606.3222, found: 606.3230.

##### Ethyl 5-butyryl-2-nitrobenzoate **15**

A mixture of 1.7 mL (6.0 eq, 16.7 mmol) 30% H_2_O_2_ and 10.5 mL DCM was cooled to 0 °C and 2.71 mL (7.0 eq, 19.5 mmol) trifluoroacetic anhydride were added slowly. At room temperature, a solution of 656 mg (1.0 eq, 2.79 mmol) ethyl 2-amino-5-butyrylbenzoate (**14**) in 3.5 mL DCM was added slowly. After stirring for 20 h, the solution was cooled to 0 °C, 10 mL saturated Na_2_SO_3_ solution was added and the mixture was extracted three times with 20 mL DCM. The combined organic phases were dried over Na_2_SO_4_, filtered and the solvent was removed at reduced pressure. The crude product was purified by flash chromatography using a gradient from 0 to 10% ethyl acetate in cyclohexane yielding 294 mg (40%) of the nitro compound as a pale yellow oil.

**^1^H NMR** (400 MHz, CDCl_3_): δ = 8.30 (d, *J* = 1.9 Hz, 1H), 8.17 (dd, *J* = 8.4, 1.9 Hz, 1H), 7.93 (d, *J* = 8.4 Hz, 1H), 4.42 (q, *J* = 7.1 Hz, 2H), 2.99 (t, *J* = 7.2 Hz, 2H), 1.79 (m, 2H), 1.37 (t, *J* = 7.1 Hz, 3H), 1.02 (t, *J* = 7.4 Hz, 3H) ppm. **^13^C NMR** (101 MHz, CDCl_3_): δ = 197.7, 164.6, 150.7, 140.1, 131.3, 129.9, 128.0, 124.4, 63.0, 41.0, 17.5, 13.9, 13.8 ppm. **HR-MS** (ESI) *m*/*z* calcd. for C_13_H_15_NNaO_5_^+^: 288.0842, found: 288.0847.

##### Ethyl 5-(1,1-diethoxybutyl)-2-nitrobenzoate **16**

To a solution of 265 mg (1.0 eq, 1.00 mmol) **15** and 1.66 mL (10.0 eq, 9.99 mmol) triethyl orthoformate in 5.0 mL ethanol were added 71 µL (1.0 eq, 1.00 mmol) acetyl chloride. After stirring for 24 h at reflux, the solution was cooled to room temperature, 5 mL saturated NaHCO_3_ solution was added and the mixture was extracted three times with 20 mL DCM. The combined organic phases were dried over Na_2_SO_4_, filtered and the solvent was removed at reduced pressure. The crude product was purified by flash chromatography using a gradient from 0 to 50% ethyl acetate in cyclohexane yielding 201 mg (58%) of the ketal as a pale yellow oil.

**^1^H NMR** (400 MHz, CDCl_3_): δ = 7.88 (d, *J* = 8.4 Hz, 1H), 7.84 (d, *J* = 1.9 Hz, 1H), 7.72 (dd, *J* = 8.5, 1.9 Hz, 1H), 4.40 (q, *J* = 7.1 Hz, 2H), 3.43 (dq, *J* = 9.4, 7.0 Hz, 2H), 3.30 (dq, *J* = 9.4, 7.1 Hz, 2H), 1.88 (m, 2H), 1.37 (t, *J* = 7.2 Hz, 3H), 1.20 (t, *J* = 7.1 Hz, 6H), 0.93 (m, 2H), 0.78 (t, *J* = 7.2 Hz, 3H) ppm. **^13^C NMR** (101 MHz, CDCl_3_): δ = 165.9, 148.5, 147.3, 130.2, 128.5, 127.9, 123.7, 102.6, 62.7, 56.8, 40.2, 16.7, 15.3, 14.0, 13.9 ppm. **HR-MS** (ESI) *m*/*z* calcd. for C_17_H_25_NNaO_6_^+^: 362.1574, found: 362.1585.

##### 5-(1,1-Diethoxybutyl)-2-nitrobenzohydrazide **17**

Compound **17** was prepared according to GP3 from 188 mg (1.0 eq, 0.554 mmol) **16**, 5.0 mL methanol and 538 µL (20.0 eq, 11.1 mmol) hydrazine hydrate. The solution was stirred for three days at room temperature. Subsequently, 30 mL water was added and the mixture was extracted five times with 20 mL DCM. The crude product yielded 177 mg (98%) of the hydrazide as a pale yellow oil, which was used without further purification.

**^1^H NMR** (300 MHz, CDCl_3_): δ = 8.04 (d, *J* = 8.5 Hz, 1H), 7.69 (dd, *J* = 8.5, 1.9 Hz, 1H), 7.65 (d, *J* = 1.8 Hz, 1H), 7.26 (s, 1H), 4.17 (s, 2H), 3.34 (ddq, *J* = 41.3, 9.3, 7.1 Hz, 4H), 1.86 (m, 2H), 1.19 (t, *J* = 7.0 Hz, 6H), 0.91 (m, 2H), 0.76 (t, *J* = 7.2 Hz, 3H) ppm. **^13^C NMR** (101 MHz, CDCl_3_): δ = 168.0, 149.4, 145.9, 130.7, 129.6, 127.8, 124.5, 102.5, 56.8, 40.2, 16.8, 15.3, 14.0 ppm. **HR-MS** (ESI) *m*/*z* calcd. for C_15_H_23_N_3_NaO_5_^+^: 348.1530, found: 348.1528.

##### 2-Cyclopropyl-9-[5-(1,1-diethoxybutyl)-2-nitrophenyl]-6-methylpyrido[3,2-e][1,2,4]triazolo[4,3-a]pyrazine **18**

Compound **18** was prepared according to GP4 from 156 mg (0.478 mmol) **17**, 4.8 mL ACN, 429 µL (2.87 mmol) DBU, 99.8 mg (0.95 eq, 0.454 mmol) 3-chloro-6-cyclopropyl-2-methylpyrido[2,3-*b*]pyrazine, 251 mg (0.956 mmol) PPh_3_ and 226 mg (0.956 mmol) C_2_Cl_6_, using isocratic conditions at 35% ethyl acetate in cyclohexane for flash chromatography and yielding 104 mg (46%) of the cyclized product as a yellow solid.

**^1^H NMR** (300 MHz, CDCl_3_): δ = 8.45 (dd, *J* = 8.3, 0.7 Hz, 1H), 8.13 (d, *J* = 8.3 Hz, 1H), 7.90 (m, 2H), 7.39 (d, *J* = 8.3 Hz, 1H), 3.41 (m, 4H), 3.07 (s, 3H), 1.90 (m, 3H), 1.19 (t, *J* = 7.0 Hz, 6H), 1.03 (m, 2H), 0.79 (t, *J* = 7.3 Hz, 5H), 0.36 (br m, 2H) ppm. **^13^C NMR** (101 MHz, CDCl_3_): δ = 163.5, 152.3, 149.4, 147.2, 147.1, 146.5, 137.7, 137.3, 132.2, 129.8, 129.1, 124.9, 124.9, 122.9, 102.6, 56.9, 40.3, 21.2, 17.3, 16.9, 15.3, 14.0, 11.3 ppm. **HR-MS** (ESI) *m*/*z* calcd. for C_26_H_31_N_6_O_4_^+^: 491.2401, found: 491.2397.

##### 1-[3-(2-Cyclopropyl-6-methylpyrido[3,2-e][1,2,4]triazolo[4,3-a]pyrazin-9-yl)-4-nitrophenyl]butan-1-one **19**

A mixture of 77.8 mg (1.0 eq, 0.159 mmol) **18** and 5.4 mg (18 mol%, 0.0285 mmol) TsOH · H_2_O in 10 mL acetone was stirred 20 h at room temperature. The solvent was removed at reduced pressure and the residue was purified by flash chromatography using a gradient from 0 to 100% ethyl acetate in cyclohexane yielding 62 mg (94%) of the butyrophenone as a yellow solid.

**TLC**: R_f_ = 0.54 (MeOH/DCM = 5/95). **HPLC**: *t*_R_ = 16.86 min (>99.6 %). **^1^H NMR** (400 MHz, CDCl_3_): δ = 8.53 (d, *J* = 8.6 Hz, 1H), 8.36 (dd, *J* = 8.6, 2.0 Hz, 1H), 8.28 (d, *J* = 2.0 Hz, 1H), 8.15 (d, *J* = 8.3 Hz, 1H), 7.37 (d, *J* = 8.3 Hz, 1H), 3.07 (s, 3H), 3.02 (t, *J* = 7.2 Hz, 2H), 1.89 (tt, *J* = 8.1, 4.7 Hz, 1H), 1.80 (m, 2H), 1.00 (t, *J* = 7.4 Hz, 3H), 0.83 (m, 2H), 0.30 (br s, 2H) ppm. **^13^C NMR** (101 MHz, CDCl_3_): δ = 197.9, 163.7, 152.3, 150.1, 146.6, 146.1, 140.6, 137.5, 137.4, 133.2, 130.7, 129.2, 125.8, 125.5, 122.7, 41.2, 21.2, 17.5, 17.3, 13.8, 11.7 ppm. **HR-MS** (ESI) *m*/*z* calcd. for C_22_H_21_N_6_O_3_^+^: 417.1670, found: 417.1674.

### 3.2. Radiochemistry

#### 3.2.1. General

No-carrier-added [^18^F]fluoride was produced via the [^18^O(p,n)^18^F] nuclear reaction by irradiation of an [^18^O]H_2_O target (Hyox 18 enriched water, Rotem Industries Ltd., Israel) on a Cyclone 18/9 (iba RadioPharma Solutions, Belgium) with fixed energy proton beam using Nirta [^18^F]fluoride XL target.

Radio thin layer chromatography (radio-TLC) was performed on silica gel (Polygram^®^ SIL G/UV_254_) pre-coated plates with a mixture of dichloromethane/methanol 10/1 (*v*/*v*) as eluent. The plates were exposed to storage phosphor screens (BAS-IP MS 2025, FUJIFILM Co., Tokyo, Japan) and recorded using the Amersham Typhoon RGB Biomolecular Imager (GE Healthcare Life Sciences, Freiburg, Germany). Images were quantified with the ImageQuant TL8.1 software (GE Healthcare Life Sciences).

Analytical chromatographic separations (radio-HPLC) were performed on a JASCO (JASCO Deutschland GmbH, Pfungstadt, Germany) LC-2000 system, incorporating a PU-2080*Plus* pump, AS-2055*Plus* auto injector (100 μL sample loop), and a UV-2070*Plus* detector coupled with a gamma radioactivity HPLC detector (Gabi Star, raytest Isotopenmessgeräte GmbH, Straubenhardt, Germany). Data analysis was performed with the Galaxie chromatography software (Agilent Technologies) using the chromatograms obtained at 254 nm. A Reprosil-Pur C18-AQ column (250 × 4.6 mm; 5 µm; Dr. Maisch HPLC GmbH; Ammerbuch-Entingen, Germany) with ACN/20 mM NH_4_OAc aq. (pH 6.8) as eluent mixture and a flow of 1.0 mL/min was used (gradient: eluent A 10% ACN/20 mM NH_4_OAc aq.; eluent B 90% ACN/20 mM NH_4_OAc aq.; 0–5 min 100% A, 5–20 min up to 100% B, 20–25 min 100% B, 25–26 min up to 100% A, 26–30 min 100% A). The ammonium acetate concentration stated as 20 mM NH_4_OAc aq. corresponds to the concentration in the aqueous component of an eluent mixture.

The molar activities were determined on the basis of a calibration curve carried out under isocratic HPLC conditions (42% ACN/20 mM NH_4_OAc_aq_; Reprosil-Pur C18-AQ, 250 × 4.6 mm) using chromatograms obtained at 230 nm as a wavelength with high absorption.

#### 3.2.2. Manual Copper-Mediated Radiofluorination Experiments

##### (a) Experiments with Azeotropic Drying

No carrier added [^18^F]fluoride in 1.0 mL of water was trapped on a Sep-Pak Accell Plus QMA carbonate Plus light cartridge (Waters GmbH, Eschborn, Germany) which was preconditioned by rinsing with 5 mL of an aqueous 0.5 M solution of NaHCO_3_ and 10 mL water. For labeling reactions with the [^18^F]F^−^/K_222_/K_2_CO_3_ complex, the activity was eluted with 400 µL of an aqueous solution of potassium carbonate (K_2_CO_3_, 1.8 mg, 13 µmol) into a 4 mL V-vial containing Kryptofix 2.2.2 (K_2.2.2_, 11 mg, 29 µmol) in 1 mL of ACN. The aqueous [^18^F]fluoride was dried by azeotropic distillation under vacuum and nitrogen flow within 7–10 min using a single mode microwave (75 W, at 50–60 °C, power cycling mode; Discover PETWave from CEM corporation, USA). Two aliquots of ACN (2 × 1.0 mL) were added during the drying procedure and the final complex was dissolved in 800 µL of DMF and divided in two portions ready for labeling. When [^18^F]TBAF was synthesized, the aqueous [^18^F]fluoride (200–400 µL) was directly added to 100 µL of an 0.075 M solution of TBAHCO_3_ (ABX advanced biochemical compounds GmbH, Radeberg, Germany) dissolved in 1 mL ACN and dried by azeotropic distillation as described above. The dried [^18^F]TBAF was then dissolved in 400 µL of the respective solvent mixture (DMA/*n*-butanol or DMA/*tert*-butanol 2/1 *v*/*v*) ready for labeling. Both fluorination agents were treated with the respective amount of [Cu(OTf)_2_(py)_4_] and stirred for 2 min at room temperature. Afterwards, the boronic acid pinacol ester precursor **13** (2 mg, 3.4 µmol) was dissolved in 400 µL of the appropriate solvent and added to the solution of copper catalyst and fluorination agent. The ^18^F-labelings were usually performed at 110 °C and in two experiments at 130 and 140 °C to investigate the influence of higher temperatures. To analyze the reaction mixture and to determine radiochemical yields, samples were taken for radio-TLC and radio-HPLC at different time points (5, 10, 15, 20 min).

##### (b) Experiments without Azeotropic Drying

No carrier added [^18^F]fluoride in 1.0 mL of water was trapped on a Chromafix^®^ 30 PS-HCO_3_^−^ cartridge (ABX, Radeberg, Germany) which was preconditioned by rinsing with 5 mL of an aqueous 0.5 M solution of NaHCO_3_ and 10 mL water. After loading, the cartridge was washed with 2.0 mL of anhydrous methanol and dried with a stream of nitrogen for 3 min. The activity was then eluted with 20 mg DMAPOTf (synthesized according to the procedure described by Zhang et al. [35]) dissolved in 500 µL of anhydrous methanol achieving activity recoveries of 85 %. The methanol was evaporated under a stream of nitrogen at 60 °C. The dry [^18^F]DMAPF was then dissolved in 800 µL of the respective solvent (DMA or DMI) and divided into two portions. One portion was used for labeling the boronic acid pinacol ester precursor **13** (2 mg, 3.4 µmol in 300 µL solvent) and the other portion for the reference reaction with 4-biphenylboronic acid pinacol ester (2 mg, 7 µmol in 300 µL solvent; Sigma-Aldrich Chemie GmbH, Taufkirchen, Germany). Before addition of the precursor solutions, the [^18^F]DMAPF was treated with the copper catalyst [Cu(OTf)_2_(py)_4_] (in a molar ratio of 1.5 to precursor) and the mixtures were stirred for 2 min at room temperature. The ^18^F-labelings were performed at 115 °C. To determine radiochemical yields, samples were taken for radio-TLC and radio-HPLC at different time points (5, 10, 15, 20 min).

#### 3.2.3. Manual Aromatic Nucleophilic Substitution Reactions Experiments

No carrier added [^18^F]fluoride (500–800 MBq) in 1.0 mL of water was trapped on a Sep-Pak^®^ Accell QMA light cartridge (Waters GmbH, Eschborn, Germany). The activity was eluted with 300 µL of an aqueous solution of potassium carbonate (K_2_CO_3_, 1.8 mg, 13 µmol) into a 4 mL V-vial containing Kryptofix 2.2.2 (K_2.2.2_, 11 mg, 29 µmol) in 1 mL of ACN. For preparation of [^18^F]TBAF, [^18^F]fluoride was placed into the V-vial containing 100 µL of an aqueous TBAHCO_3_ solution (0.075 M, ABX, Radeberg, Germany) and 1 mL of ACN. The aqueous [^18^F]fluoride was dried by azeotropic distillation as described above and the respective final fluorination agent was dissolved in 1.0 mL of labeling solvent and divided in two portions. Thereafter, a solution of 1–2 mg of precursor **19** dissolved in 300 µL of the appropriate solvent was added, and ^18^F-labeling was performed at different temperatures in dependence of the solvent used. To analyze the reaction mixture and to determine radiochemical yields, samples were taken for radio-TLC and radio-HPLC at different time points (5, 10, 15, 20 min).

#### 3.2.4. Automated Radiosynthesis of **[^18^F]11**

Remote controlled radiosynthesis of **[^18^F]11** was performed using a TRACERlab FX2 N synthesizer (GE Healthcare, Waukesha, USA) equipped with a Laboport vacuum pump N810.3FT.18 (KNF Neuburger GmbH, Freiburg, Germany), a BlueShadow UV detector 10D (KNAUER GmbH, Berlin, Germany) and the TRACERlab FX Software.

**Figure 8:**
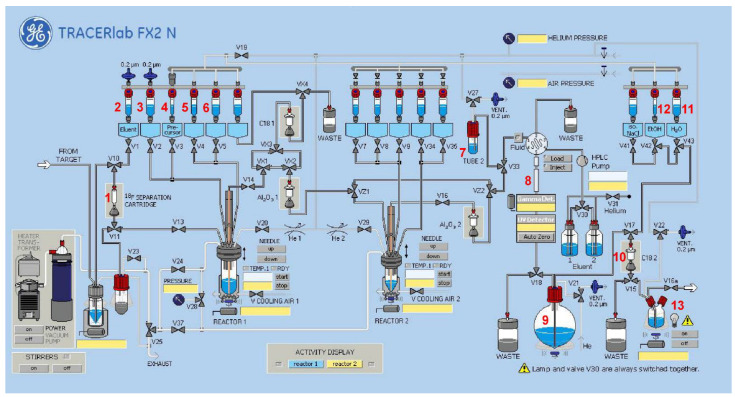
Scheme of the synthesis module TRACERlab FX2 N for the radiosynthesis of **[^18^F]11**. (1) Sep-Pak^®^ Accell QMA light, (2) 150 µL 0.075 M TBAHCO_3_, 700 µL ACN, 300 µL water, (3) 2.5 mL ACN, (4) 1.0 mg of **19** in 800 µL ACN), (5) 24 µL of 2M NaBH_4_ in 400 µL *n*-butanol, (6) 2.0 mL of 20 mM aqu. ammonium formate, 0.5 mL water and 0.5 mL ACN, (7) injection vial, (8) Reprosil-Pur C18-AQ 250 × 10 mm (50% ACN/20 mM NH_4_OAc_aq._, flow 6.5 mL/min), (9) 40 mL water, (10) Sep-Pak^®^ C18 light, (11) 2 mL water, (12) 1.2 mL EtOH, (13) product vial.

[^18^F]Fluoride (6–8 GBq) was trapped on a Sep-Pak^®^ Accell QMA light cartridge (Figure 8, entry **1**) and eluted into the reactor with 1150 µL of an aqueous ACN solution of TBAHCO_3_ (150 µL 0.075 M TBAHCO_3_, 700 µL ACN, 300 µL water, entry **2**). After azeotropic distillation for 4 min at 50 °C, 2.5 mL of ACN was added and the mixture was further azeotropically dried for 7 min at 70 °C. Thereafter, 1.0 mg (2.4 µmol) of the nitro precursor **19** dissolved in 800 µL of ACN (entry **4**) was added, and the reaction mixture was stirred at 110 ⁰C for 12 min. After cooling to 30 °C, 24 µL (48 µmol) of a 2M sodium borhydride solution (2M NaBH_4_ in triethylene glycol dimethyl ether, Sigma-Aldrich Chemie GmbH, Taufkirchen, Germany) in 400 µL *n*-butanol was added (entry **5**) and reduction of the keton function was performed within 5 min at 30 °C. After cooling, the reaction mixture was diluted with 2.0 mL of 20 mM aqu. ammonium formate, 0.5 mL water and 0.5 mL ACN (entry **6**) and transferred into the injection vial (entry **7**). Semi-preparative radio-HPLC was performed using a Reprosil-Pur C18-AQ column (250 × 10 mm; 10 µm; Dr. Maisch HPLC GmbH, Germany) with a solvent composition of 50% ACN/20 mM NH_4_OAc_aq._ at a flow rate of 6.5 mL/min (entry **8**). **[^18^F]11** was collected in the dilution vessel (entry **9**) previously loaded with 40 mL of H_2_O. Final purification was performed by passing the solution through a Sep-Pak^®^ C18 light cartridge (entry **10**), followed by washing with 2 mL of water (entry **11**) and elution of **[^18^F]11** with 1.2 mL of EtOH (entry **12**) into the product vial (entry **13**). The ethanolic solution was transferred out of the hot cell and the solvent was reduced under a gentle argon stream at 75 ⁰C to a final volume of 10–50 µL. Afterwards the radiotracer was diluted in isotonic saline to obtain a final product containing 10% of EtOH (*v*/*v*).

#### 3.2.5. Determination of Stability and logD Value

The determination of stability and logD value has been performed according to the procedures described by Sadeghzadeh and Wenzel et al. [45].

### 3.3. Biology

#### 3.3.1. In Vitro Autoradiography

Frozen sagittal 12 µm brain sections obtained from female SPRD and male Wistar-Han rats (10–12 weeks old) were thawed, dried in a stream of cold air, and pre-incubated for 15 min with incubation buffer (50 mM TRIS-HCl, pH 7.4; supplemented with 1 mM MgCl_2_, 1 mM CaCl_2_, 2 mM KCl, and 2 U/mL Adenosine deaminase) at ambient temperature. Afterwards, the buffer was decanted and the cryosections dried again in a stream of cold air before covering with the radioligand in incubation buffer (~0.1 MBq **[^18^F]11**/mL; A_m_ at the time of the incubation ~10 GBq/µmol) for incubation at ambient temperature for 90 min. For the determination of the non-specific binding the radioligand solution was supplemented with 1 µM of the PDE2-specific ligands BAY-60-7550 (IC50 for human PDE2A = 4.7 nM, for bovine PDE2A = 2.0 nM [46]) or PF-0518099 (IC50 for PDE2A = 1.6 nM [6]).

For the confirmation of the saturability of the binding sites as well as for the homologous competition experiment performed to estimate the *K*_D_ of **11**, the radioligand solution was supplemented with different concentrations of non-labeled **11** (0.1 nM–1 µM). The incubation was terminated by pouring off the incubation solution and washing the slides twice with ice-cold buffer (50 mM TRIS-HCl, pH 7.4/4 °C) for 2 min and rinsing by dipping in ice-cold demineralised water. Immediately afterwards, the sections were dried in a stream of cold air and exposed along with appropriate radioactive standards (1 µL of serial dilutions of the radioligand in incubation buffer, pipetted on a microscopic glass slide and air dried) to an ^18^F-sensitive image plate for 120 min. Finally, the imaging plate was scanned at the highest possible resolution (pixel size 12.6 µm × 12.6 µm) in a phosphor imager (HD-CR 35; Duerr NDT GmbH, Bietigheim Bissingen, Germany) and the digital image analysed with an image analysis program (Aida Image Analyzer v. 4.26; Elysia-raytest GmbH, Straubenhardt, Germany).

For the determination of the protein concentration, four dried 12 µm cryosections were scraped from the microscopic glass slides, transferred in an Eppendorf tube and solubilised with 200 µL of demineralised water. The protein concentration of this solution was determined by a commercial assay (Pierce™ BCA Protein Assay Kit, Thermo Fisher Scientific, Schwerte, Germany). The area of the cryosections was determined during the analysis of the digitized autoradiographic images, and the volume was calculated by multiplying the area with the thickness of the slices. Finally, the protein concentration of the tissue was calculated (37 mg protein/g wet weight).

#### 3.3.2. Metabolite Studies

Two non-anesthetized female CD-1 mice (30 and 33 g, 90 days) were injected intravenously with **[^18^F]11** (28 MBq = 94 nmol/kg and 25 MBq = 78 nmol/kg). The animals were anesthetized by isoflurane inhalation at 28 min p.i. and blood was collected from the retro-orbital plexus, followed by cervical dislocation at 30 min p.i. Immediately afterwards, the brains were isolated, washed twice with isotonic saline and stored on ice. Plasma was obtained from the whole blood sample by centrifugation (8.000 rpm, 2 min, ambient temperature). All samples were weighed and the radioactivity was measured in a dose calibrator (ISOMED 2010 Dose Calibrator, NUVIA Instruments GmbH, Dresden, Germany). Afterwards, the brains were homogenized in 1 mL demineralized water on ice (Potter S, B. Braun Biotech, Melsungen, Germany).

Metabolism in rats has been investigated in the animals applied in PET studies (protocol reported below). In brief, blood was taken from two anesthetized female SPRD rats 30 min after intravenous injection of **[^18^F]11** followed by decapitation and isolation of the brains. The blood samples and the brains were processed as described above.

Protein precipitation was performed by the addition of ice-cold ACN/H_2_O (9:1, *v*/*v*) in a ratio of 4:1 (*v*/*v*) of organic solvent to plasma or brain homogenate, respectively. The samples were vortexed for 3 min, equilibrated on ice for 3 min, and centrifuged for 10 min at 10,000 rpm and the supernatant collected. The precipitates were washed with 100 μL of the solvent mixture and subjected to the same procedure. The combined supernatants were concentrated at 70 °C under argon flow to a final volume of approximately 100 μL and analysed by analytical radio-HPLC with a gradient system (see radiochemistry general section). The peak corresponding to the radiotracer in the radio-chromatogram was identified by co-injecting the sample with the reference compound **11**. To determine the percentage of activity in the supernatants compared to total activity, aliquots of each step as well as the precipitates were quantified by an automated gamma counter (1480 WIZARD, Perkin Elmer, Turku, Finland). For plasma and brain samples recoveries of ≥91% of total activity were obtained.

#### 3.3.3. In Vivo PET Studies in CD-1 Mice and SPRD Rats

For the time of the experiments, one female CD-1 mouse (10 weeks; 30.5 g) and one female SPRD rat (9 weeks; 256 g) were kept in a dedicated climatic chamber with free access to water and food under a 12:12 h dark:light cycle at a constant temperature (24 °C). The animals were anesthetized (Anaesthesia Unit U-410, agntho’s, Lidingö, Sweden) with isoflurane (2.0%, 300 mL/min) delivered in a 60% oxygen/40% air mixture (Gas Blender 100 Series, MCQ instruments, Rome, Italy) and their body temperature maintained at 37 °C with a thermal bed system for the duration of the PET studies. A dynamic PET scan (PET/MR 1T, Mediso nanoScan^®^, Hungary) started 20 s before intravenous injection into the tail vein of [^18^F]**11** and was performed in mouse for 60 min (4.9 MBq in 150 μL isotonic saline; 20.5 nmol/kg; A_m_: 8.9 GBq/μmol, EOS) in rat for 30 min (29.7 MBq in 400 μL isotonic saline; 12 nmol/kg; A_m_: 11 GBq/μmol, EOS). Each PET image was corrected for random coincidences, dead time, scatter and attenuation (AC), based on a whole body (WB) MR scan. The reconstruction parameters for the list mode data were 3D-ordered subset expectation maximization (OSEM), 4 iterations, 6 subsets, energy window: 400–600 keV, coincidence mode: 1–5, ring difference: 81. Preceding the PET scan a T1 weighted WB gradient echo sequence (TR/TE: 20/6.4 ms, NEX: 1, FA: 25, FOV: 64 × 64 mm, Matrix: 128 × 128, STh: 0.5 mm) was performed for AC and anatomical orientation. PET Images were analyzed with PMOD (PMOD Technologies LLC, v. 4.1, Zurich, Switzerland). The respective brain regions were identified using the mouse brain atlas template Ma-Benveniste-Mirrione-T2 or the rat brain atlas template Schwarz-T2. The regional activity data (radioactivity concentration, kBq/mL) were normalized to the injected dose (MBq) and corrected for the animal weight (g) to provide standardized uptake values (SUV, g/mL). Finally, the results of the PETscans are expressed as mean SUV of the respective region of interest ROI.

## 4. Conclusions

With the aim to develop a specific radiotracer for neuroimaging of the cyclic nucleotide phosphodiesterase PDE2A by PET, a new ^18^F-labeled triazolopyridopyrazine-based PDE2A inhibitor was developed. **[^18^F]11** was obtained in a two-step one-pot radiolabeling procedure, and the establishment of an automated radiosynthesis in the TRACERlab synthesis module enabled a reproducible production with sufficient radiochemical yields and high radiochemical purity. In vitro autoradiography indicated high affinity and specific binding of [^18^F]**11** towards PDE2A in the rat brain with apparent *K*_D_ and *B*_max_ values of about 0.2 nM and 20 pmol/mg protein in the PDE2A-rich brain region nucleus caudate and putamen. In vivo PET/MR studies in mice and rat revealed a moderate brain uptake, however, no considerable accumulation of activity in PDE2A-specific regions was observed. Furthermore, in vivo metabolism studies revealed a significant fraction of brain-penetrant radiometabolites of **[^18^F]11**. Altogether, **[^18^F]11** shows excellent in vitro properties for the investigation of the enzyme PDE2A in the brain and is a suitable starting point for further developments towards a successful imaging agent for PET neuroimaging of PDE2A.

## Figures and Tables

**Table 1:** CMRF conditions and RCYs of the attemps to obtain the intermediate **[^18^F]9**.

Fluorination Agent	Entry	Solvent	13 (µmol)	Ratio 13 to [Cu(OTf)_2_(py)_4_]	RCY (%) ^a^	RCY (%) ^b^
[^18^F]TBAF	1	DMA/*tert*-BuOH ^c^	3.4	1:3.5	0.6	-
	2	DMA/*n*-BuOH ^c^	3.4	1:1	0	-
	3	DMA/*n*-BuOH ^c^	3.4	1:3.5	0	68
	4	DMA/*n*-BuOH ^c^	8.5	1:2	0	-
[^18^F]F^−^/K_222_/K_2_CO_3_	5	DMF	3.4	1:1.5	0	-
	6	DMF	3.4	5:1	0	-
[^18^F]DMAPF ^d^	7	DMA	3.4	1:1.5	0.7	54
	8	DMI	3.4	1:1.5	0	88
	9	DMI	3.4	1:0.5	0.5	-

^a^ RCY of **[^18^F]9** determined by radio-TLC of samples taken from the reaction mixture after 15 min. ^b^ RCY of 4-[^18^F]fluorobiphenyl determined by radio-TLC of samples taken from the standard reaction of 4-biphenylboronic acid pinacol ester (2 mg, 7 µmol) with [Cu(OTf)_2_(py)_4_]. ^c^ Ratio of DMA/BuOH 2/1 (*v*/*v*). ^d^ Without azeotropic drying.

**Table 2:** Radiolabeling conditions and RCYs of the attempts to obtain the intermediate **[^18^F]12**.

Fluorination Agent	Entry	Solvent	Temperature (°C)	RCY (%) ^a^
[^18^F]F^−^/K_222_/K_2_CO_3_	1	ACN	110	0
	2	DMF	150	0
	3	DMSO	150	0
[^18^F]TBAF	4	ACN	110	21
	5	ACN/DMSO ^b^	130	10
	6	*tert*-butanol	90	0
	7	DMF	160	14

^a^ RCY of **[^18^F]12** determined by radio-TLC of samples taken from the reaction mixture after 15 min. ^b^ Ratio of ACN/DMSO 1/1 (*v*/*v*).

## Data Availability

Data is contained within article and Supplementary Materials.

## Supplementary Materials

The following supporting information can be downloaded at: https://www.mdpi.com/article/10.3390/ph15101272/s1, Figures S1–S11: analytical data of organic syntheses, Figures S12–S13: radio-and UV-HPLC data of radiosyntheses, Figure S14 and Table S1: Biodistribution data.
