# Supplementary material for: Radiosynthesis and Preclinical Evaluation of an 18F-Labeled Triazolopyridopyrazine-Based Inhibitor for Neuroimaging of the Phosphodiesterase 2A (PDE2A)
Source: Pharmaceuticals (Basel). 2022 Oct 15;15(10):1272. doi: 10.3390/ph15101272 (PMC9609767; doi:10.3390/ph15101272)
Supplement: Supplementary file 1 [file pharmaceuticals-15-01272-s001.zip › pharmaceuticals-1921384-supplementary.pdf]

Supplementary Materials

to

Radiosynthesis and preclinical evaluation of an ^{18}F-labeled triazolopyridopyrazine-based inhibitor for neuroimaging of the phosphodiesterase 2A (PDE2A)

Barbara Wenzel [1], Stefan R. Fritzsche [2], Magali Toussaint [1], Detlef Briel [2], Klaus Kopka [1,3], Peter Brust [1], Matthias Scheunemann [1] and Winnie Deuther-Conrad [1]

[1] Department of Neuroradiopharmaceuticals, Institute of Radiopharmaceutical Cancer Research, Helmholtz-Zentrum Dresden-Rossendorf, Leipzig, Germany;

[2] Institute for Drug Discovery, Faculty of Medicine, Leipzig University, Leipzig, Germany;

[3] Faculty of Chemistry and Food Chemistry, School of Science, Technical University Dresden, Dresden, Germany;

Content:

1) NMR and mass spectra of inhibitor **11** and precursor compounds **13** and **19**

2) Radio- and UV-HPLC chromatograms of

i) semi-preparative separation of **[^{18}F]11** and

ii) final product **[^{18}F]11**

3) Biodistribution data of **[^{18}F]11** in mouse

1) NMR and mass spectra of inhibitor 11 and precursor compounds 13 and 19

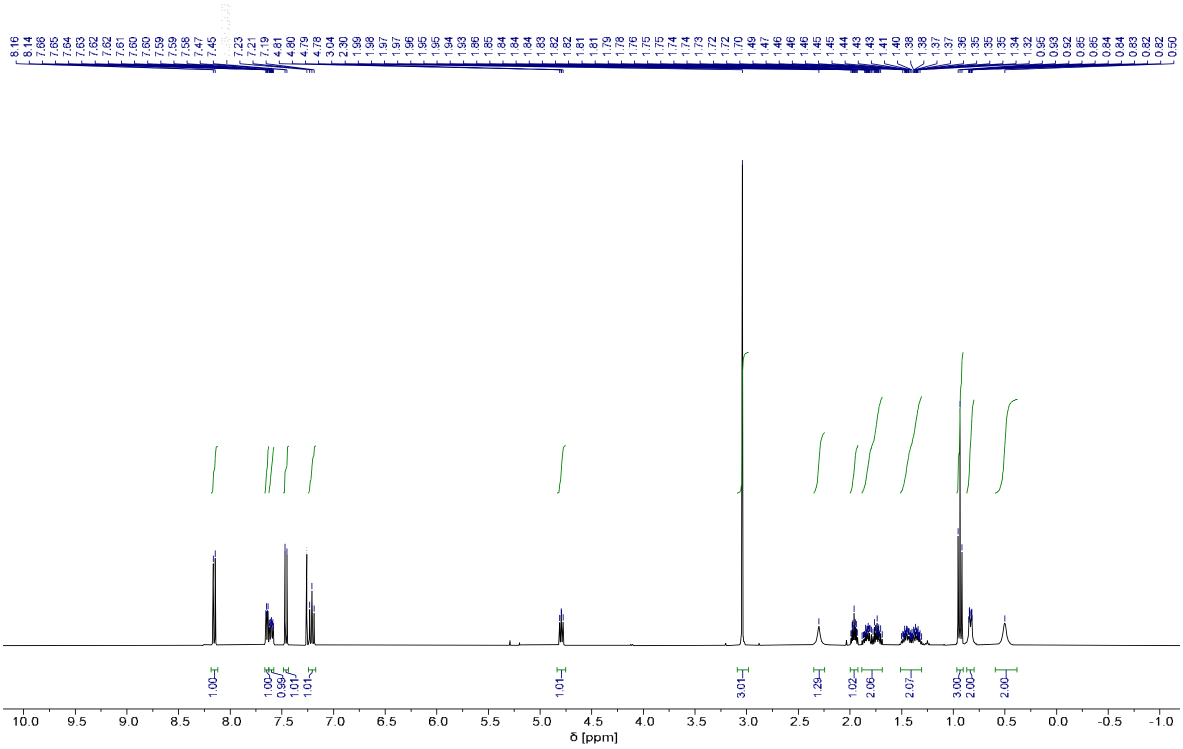

Figure S1: ^{1}H NMR spectrum (400 MHz, $CDCl_3$) of **11**

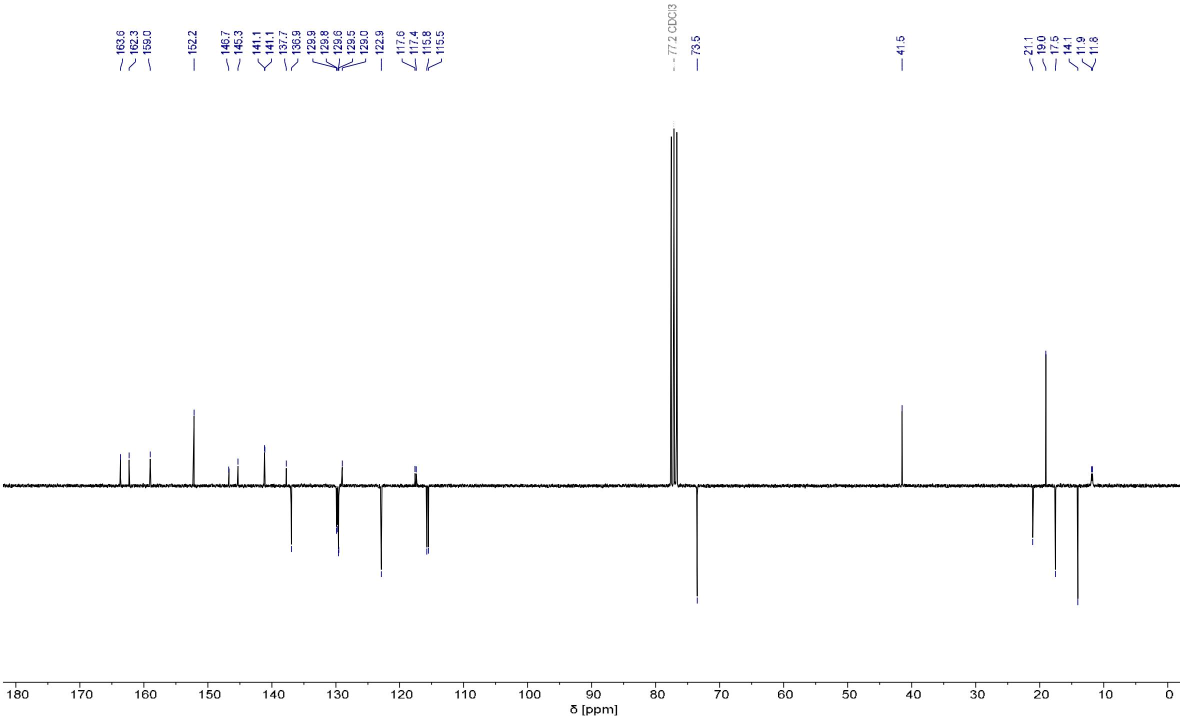

Figure S2: ^{13}C NMR spectrum (75 MHz, $CDCl_3$) of **11**

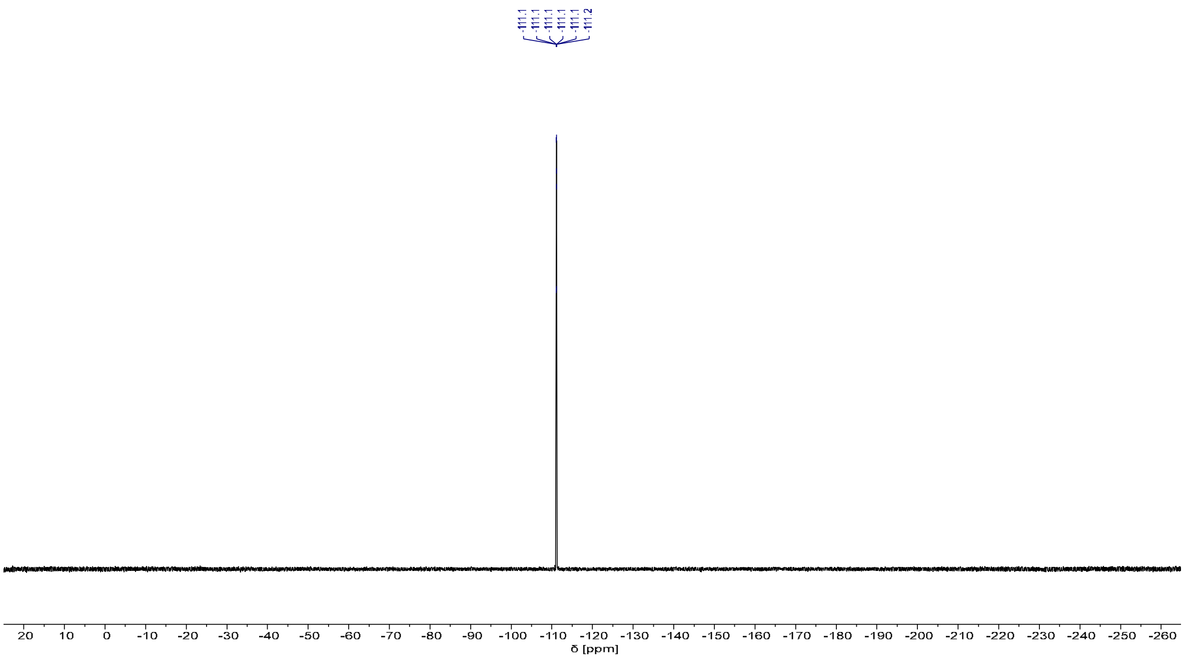

Figure S3: ^{19}F NMR spectrum (282 MHz, $CDCl_3$) of **11**

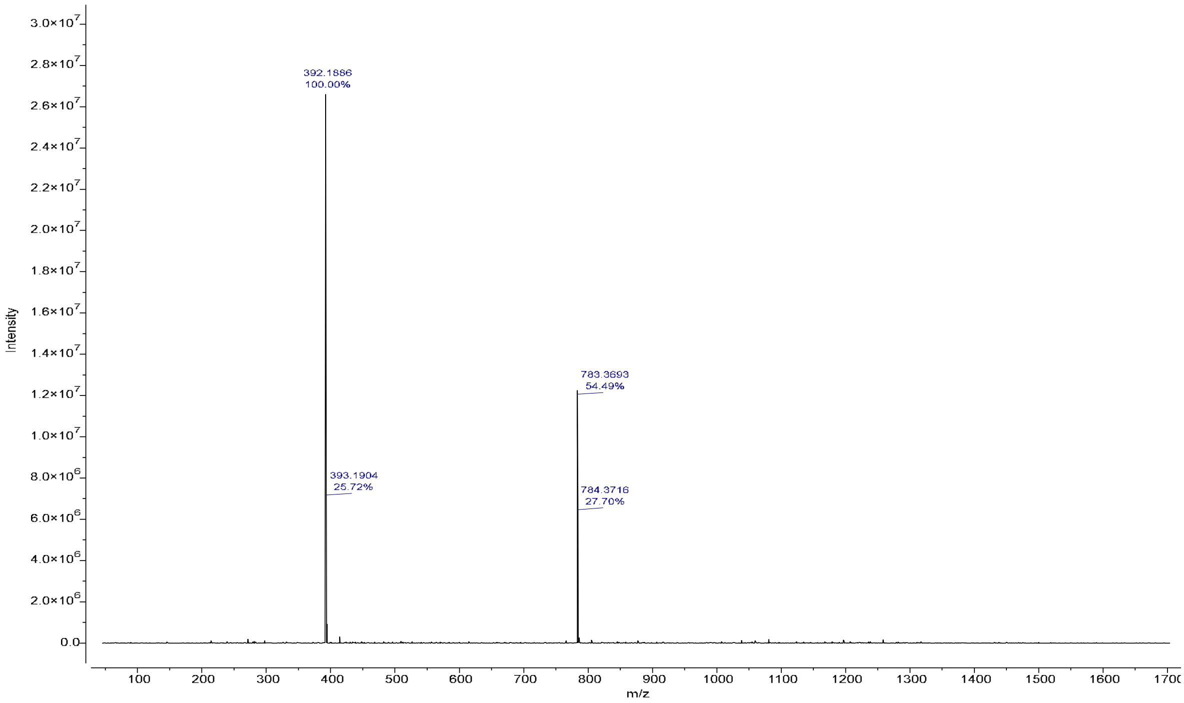

Figure S4: HR mass spectrum (ESI+) of **11**

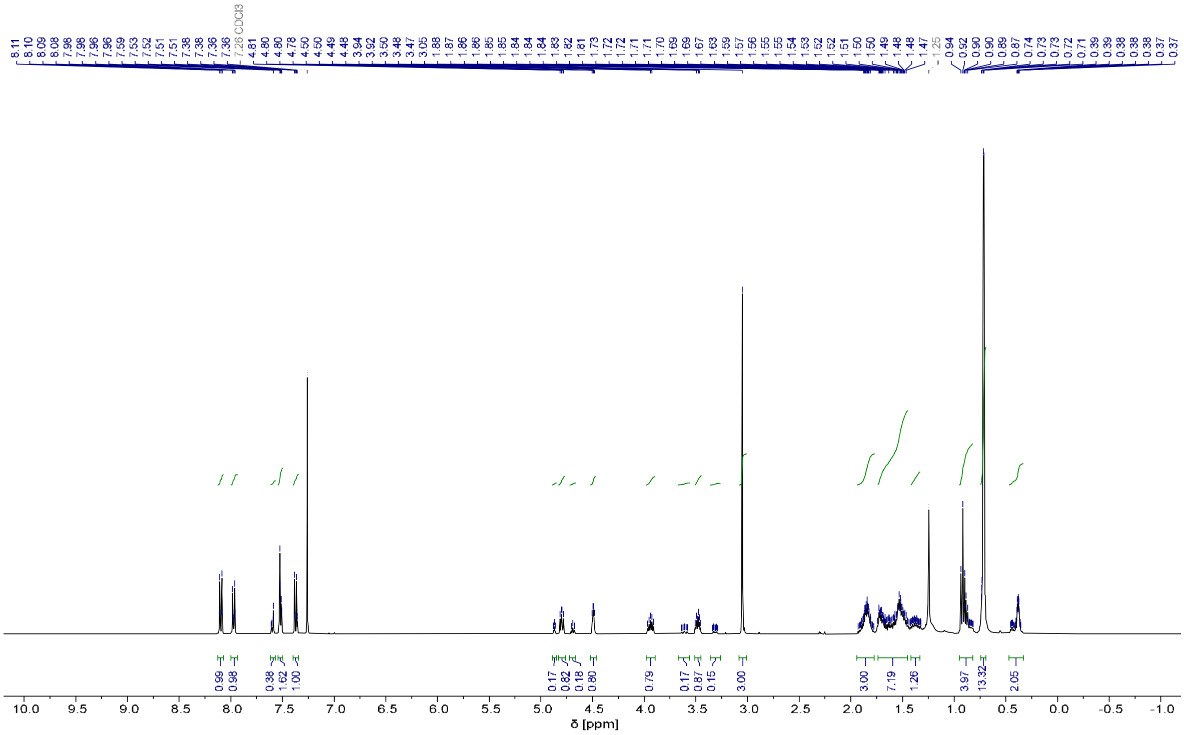

Figure S5: ^{1}H NMR spectrum (400 MHz, $CDCl_3$) of **13** (diastereomers)

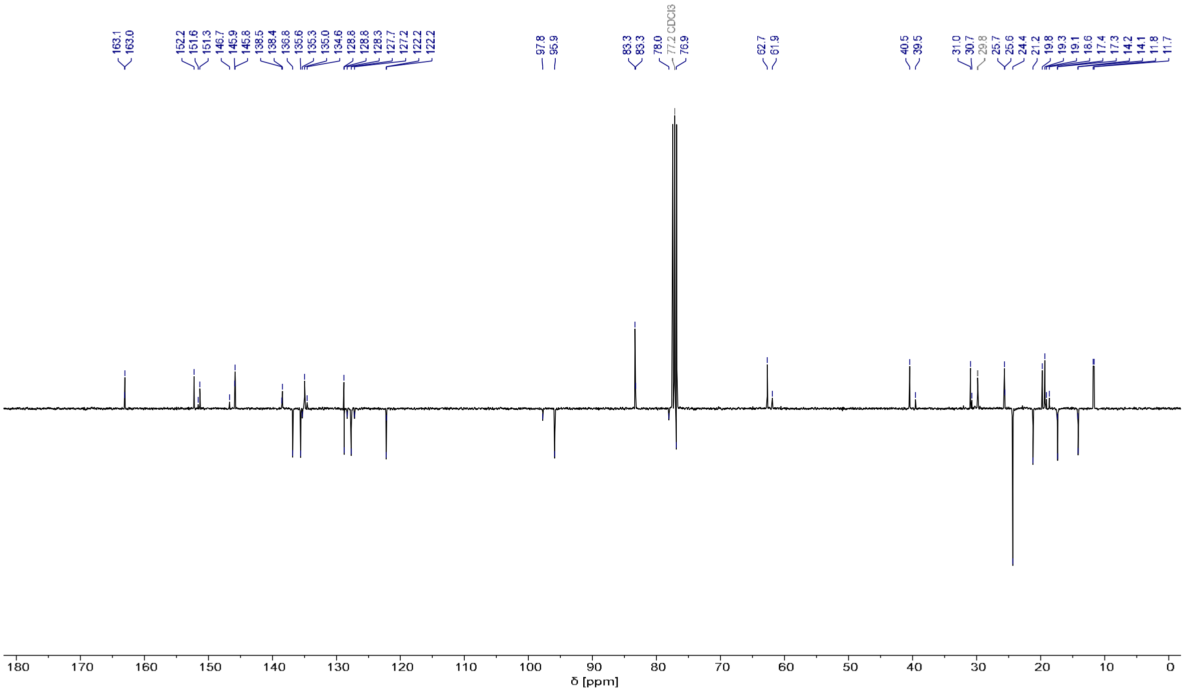

Figure S6: ^{13}CNMR spectrum (101 MHz, $CDCl_3$) of **13** (diastereomers)

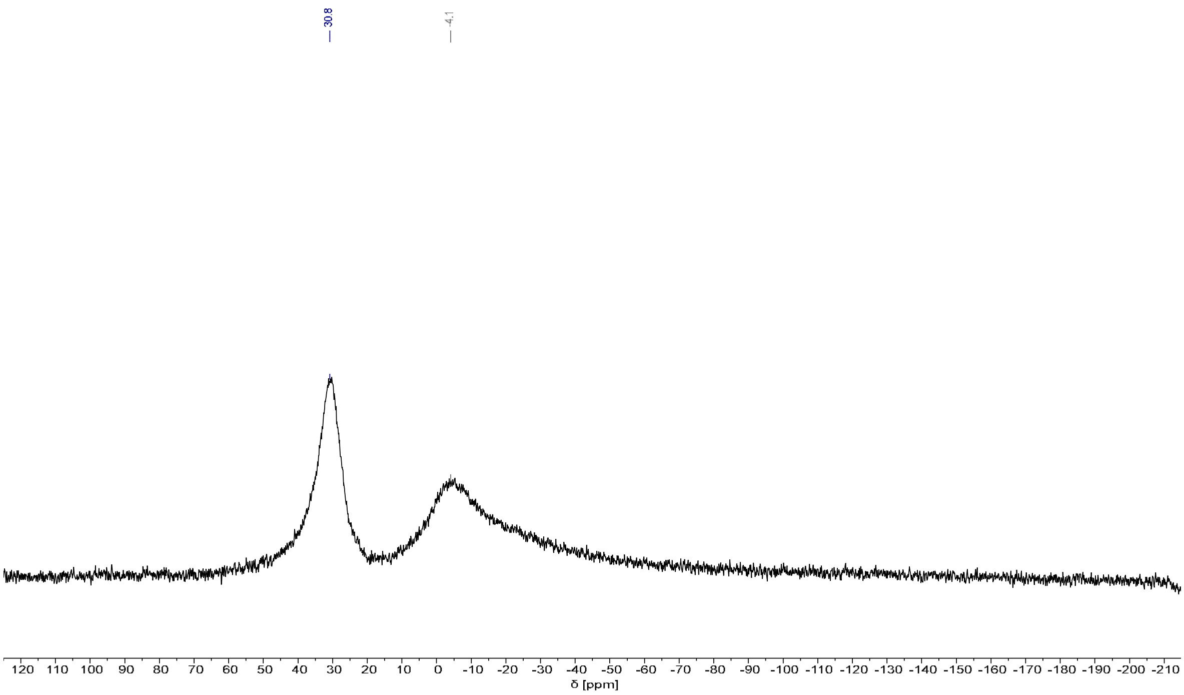

Figure S7: ^{11}B NMR spectrum (128 MHz, $CDCl_3$) of **13** (diastereomers)

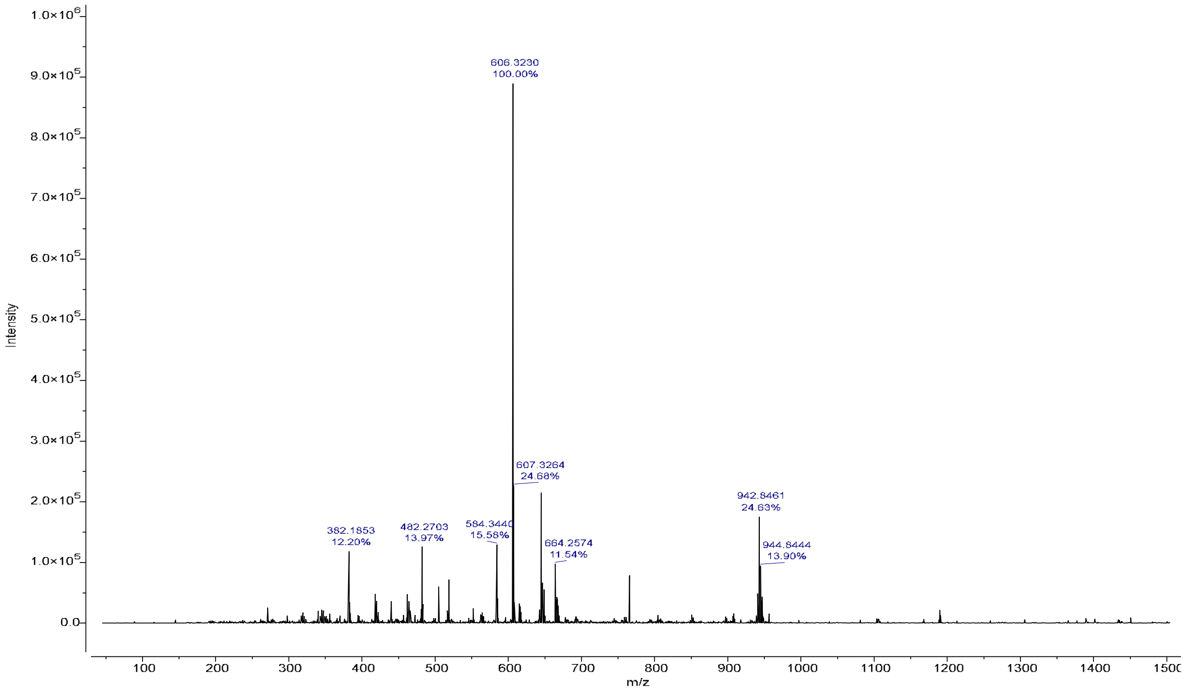

Figure S8: HR mass spectrum (ESI+) of **13**

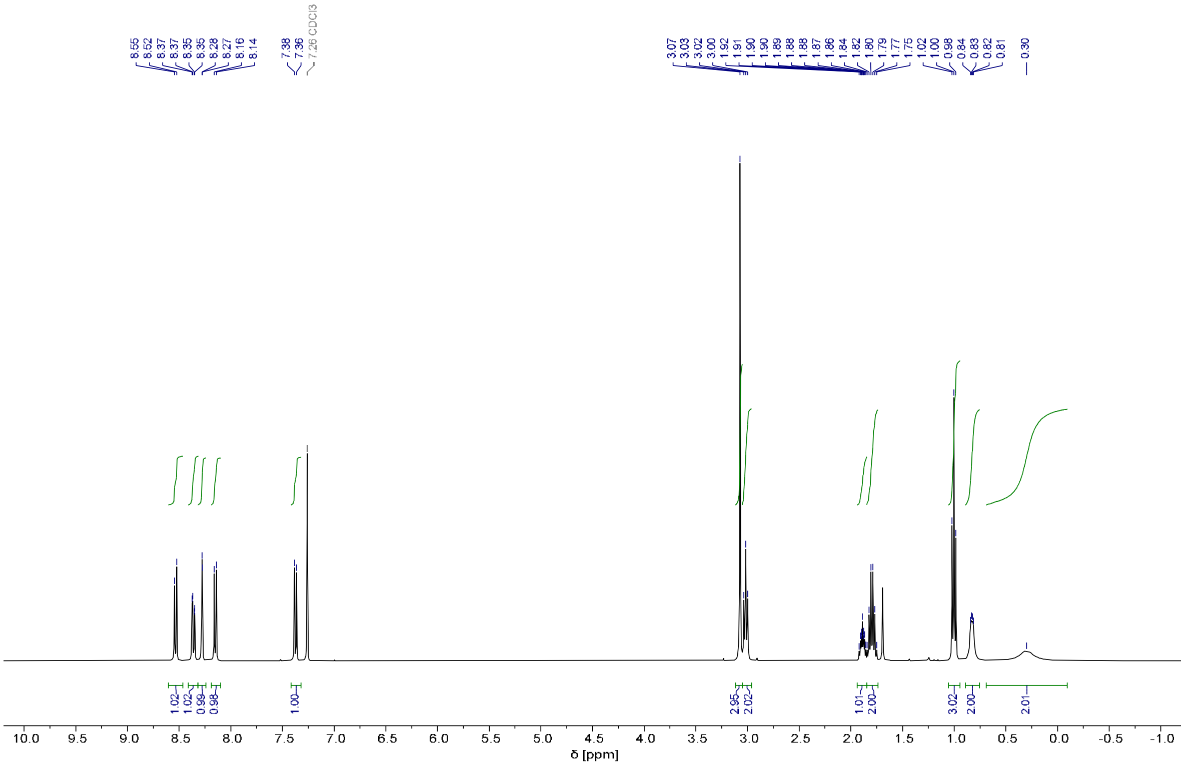

Figure S9: ^{1}H NMR spectrum (400 MHz, $CDCl_3$) of **19**

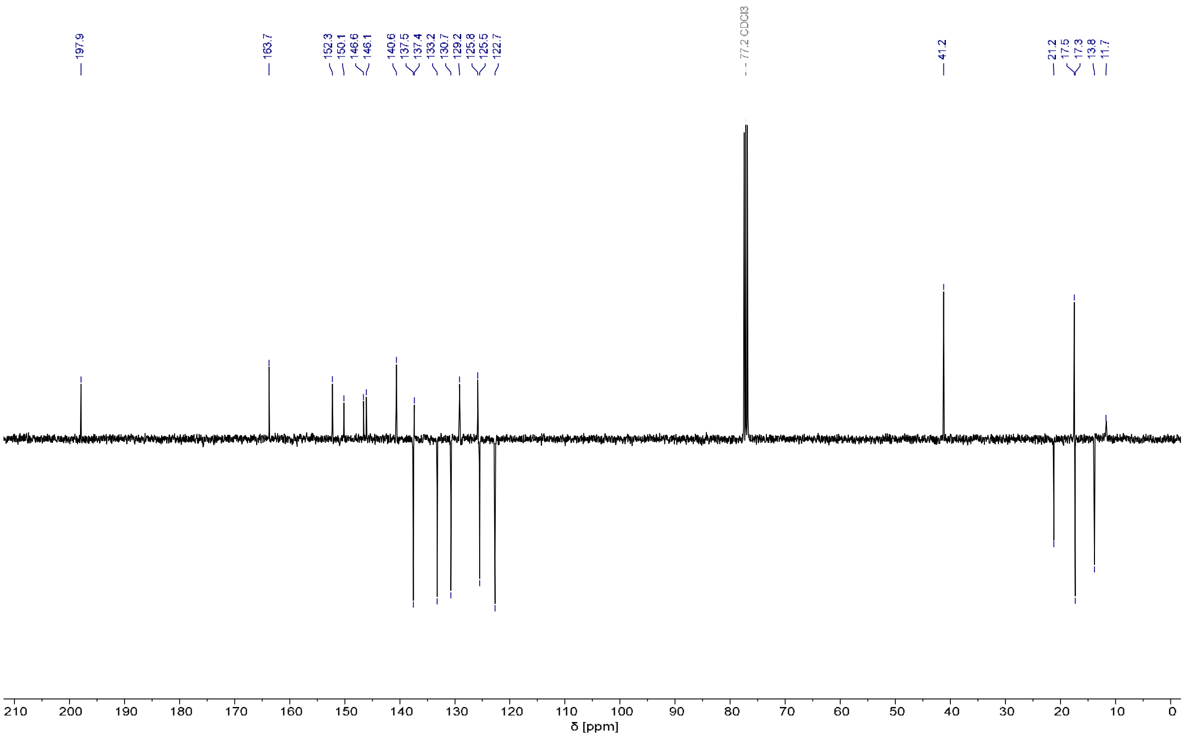

Figure S10: ^{13}C NMR spectrum (101 MHz, $CDCl_3$) of **19**

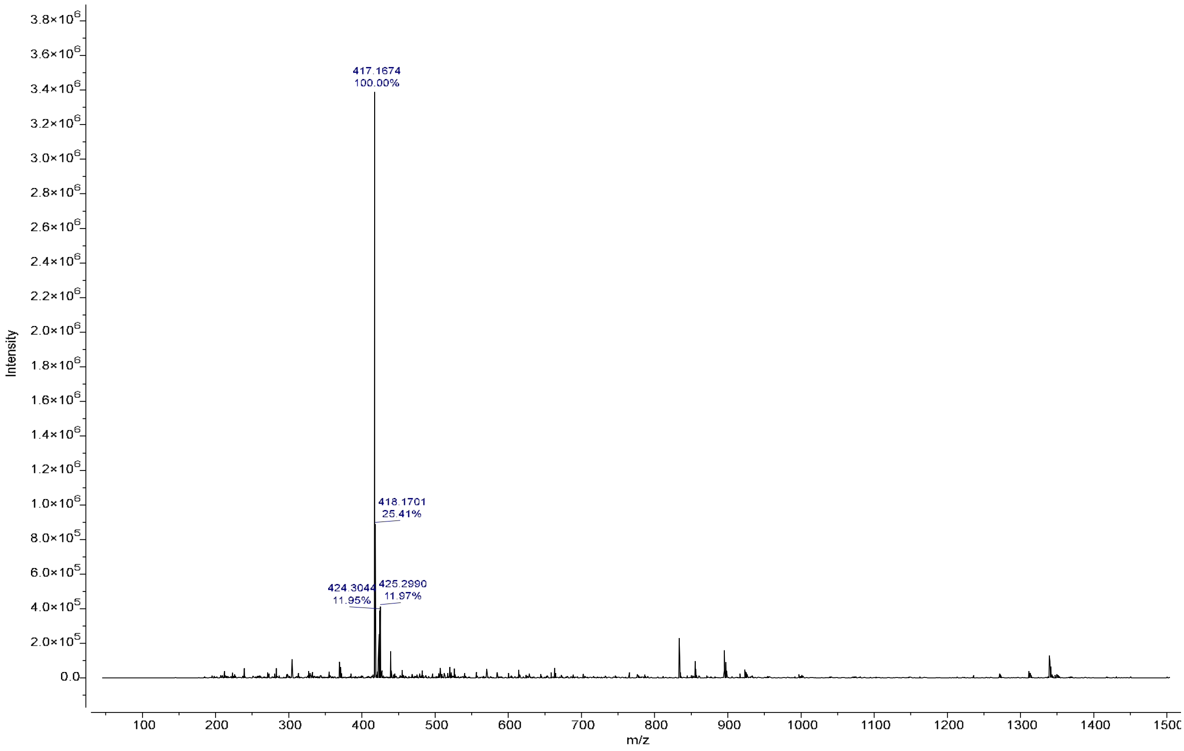

Figure S11: HR mass spectrum (ESI+) of **19**

2) Radio- and UV-HPLC chromatograms of [^{18}F]11

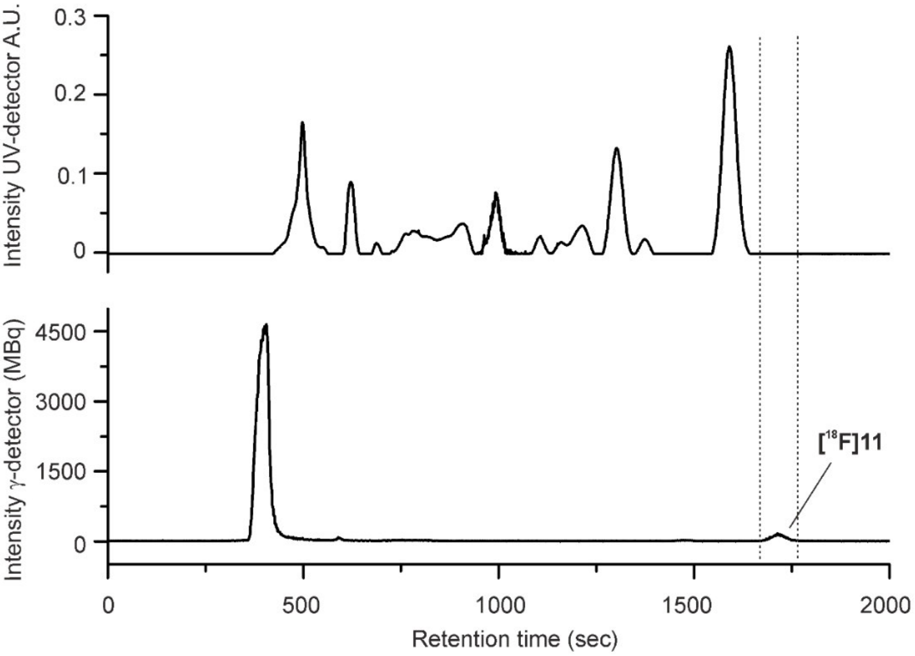

Figure S12. Exemplary UV- and radio-chromatogram of the semi-preparative HPLC separation of **[^{18}F]11**. Conditions: Reprosil-Pur C18 AQ (250 × 20 mm), 50% ACN/20 mM $NH_4OAc_{aq.}$, flow 6.5 mL/min.

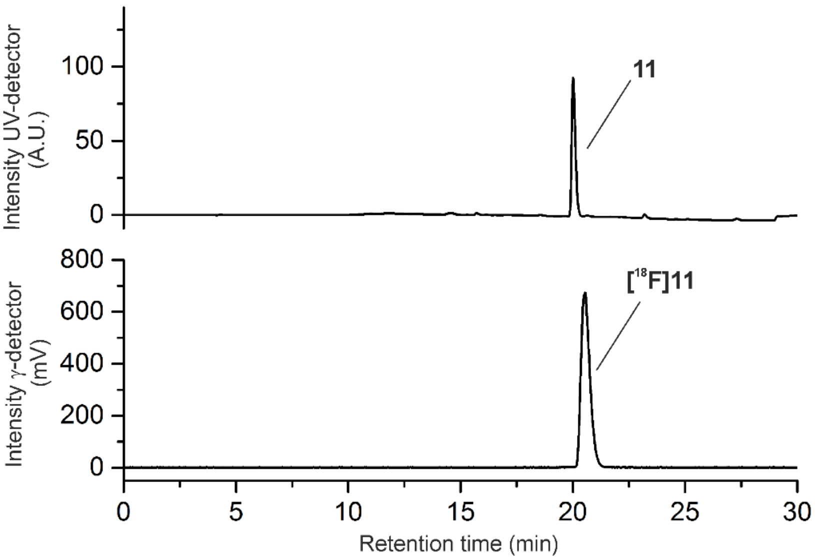

Figure S13. Exemplary UV- and radio-chromatogram of the final product of **[^{18}F]11** spiked with the nonradioactive reference compound **11**.
Conditions: Reprosil-Pur C18 AQ (250 × 4.6 mm), gradient mode ACN/20 mM $NH_4OAc_{aq.}$, flow 1.0 mL/min.

3) Biodistribution data of [18F]11 in mouse and rat

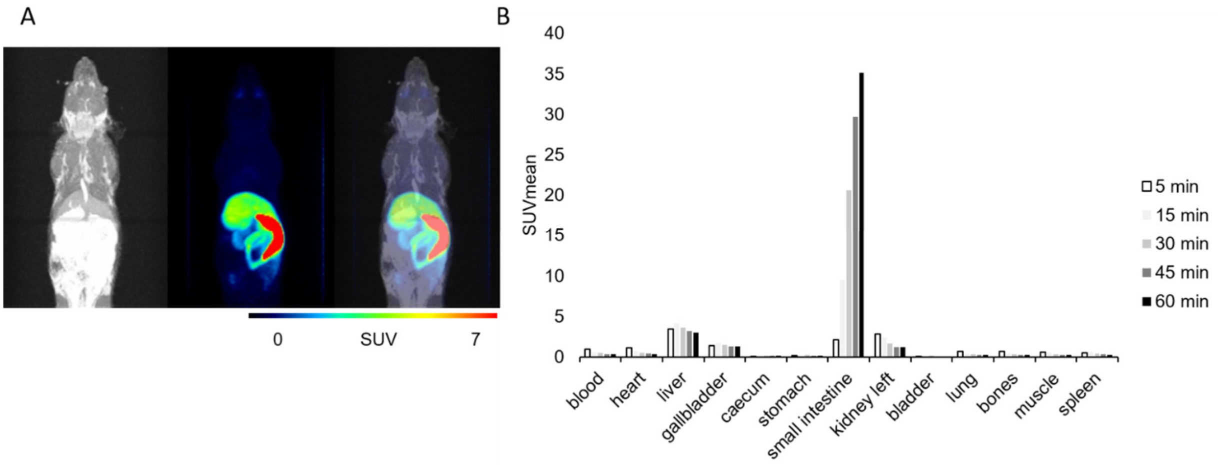

Figure S14: Maximal intensity projection [^{18}F]**11** after i.v. injection in a CD-1 mouse on MR, PET and PET/MR modalities (A). Biodistribution of [^{18}F]**11** at different time points derived from PET imaging uncorrected for metabolites (n = 1, SUV mean) (B).

Table S1: Tissue biodistribution of radioactivity at different time points after i.v. injection of [^{18}F]**11** in a CD-1 mouse based on PET data uncorrected for metabolites.

	**Uptake (SUVmean; n = 1)**				
	5 min	15 min	30 min	45 min	60 min
blood	0.93	0.56	0.46	0.34	0.32
heart	1.11	0.65	0.52	0.37	0.35
liver	3.40	4.16	3.62	3.17	3.00
gallbladder	1.41	1.78	1.46	1.32	1.34
caecum	0.10	0.12	0.11	0.11	0.16
stomach	0.15	0.18	0.19	0.17	0.13
small intestine	2.10	9.48	20.64	29.65	35.11
kidney left	2.79	2.37	1.61	1.18	1.17
bladder	0.10	0.08	0.10	0.07	0.06
lung	0.65	0.43	0.33	0.25	0.25
bones	0.71	0.47	0.31	0.23	0.20
muscle	0.63	0.49	0.35	0.26	0.21
spleen	0.48	0.59	0.45	0.30	0.24
